# Mesoporous Catalytic‐Adsorptive Nanoregulator Orchestrates Biofilm eDNA/LPS Disassembly and TLR9/TLR4 Immune Reprogramming to Resolve Diabetic Foot Infections

**DOI:** 10.1002/advs.76300

**Published:** 2026-06-26

**Authors:** Junfeng Song, Yang Song, Xirui Huang, Xingjin Li, Lifei Gao, Yixiang Wang, Tianbao Zhu, Xiaomin Li, Shuai Wang, Tiancong Zhao, Dongyuan Zhao

**Affiliations:** ^1^ Lab of Advanced Materials, College of Smart Materials and Future Energy, Department of Chemistry Fudan University Shanghai China

**Keywords:** biofilm, diabetic wound infection, eDNA, mesoporous, toll‐like receptors

## Abstract

Chronic diabetic foot infections are severely hindered by tough biofilms and a self‐fueling hyperinflammatory microenvironment, primarily orchestrated by the synergistic interplay between extracellular DNA (eDNA) and lipopolysaccharides (LPS) which facilitates the assembly of impenetrable biofilm architectures while inducing cross‐inflammatory activation through the TLR4/9 axis. Conventional therapies often fail to resolve these “dual traps” of structural resistance and immunological interference. Herein, we developed a multifunctional mesoporous nano‐regulator composed of Colistin (CT) and m‐aminophenol formaldehyde (mAPF) framework, hence termed CT/mAPF to implement a “triad‐strategy”: biofilm disruption, bacterial eradication, and debris neutralization. The CT/mAPF nano‐regulator achieves potent bactericidal activity (99.99%) and initiates ROS‐mediated oxidative fragmentation of eDNA to destroy the biofilm scaffold. Crucially, the platform effectively neutralizes LPS and degrades eDNA, leading to the simultaneous silencing of TLR4 and TLR9 signaling pathways. This dual‐targeting approach weakens the eDNA/LPS‐mediated synergistic inflammatory response, and suppresses pro‐inflammatory cytokines (*IL‐6*, *IL‐1β*, *TNF‐α*). In diabetic mouse models, CT/mAPF significantly accelerated bacterial clearance and wound closure through enhanced angiogenesis and collagen maturation. This integrated strategy resolves the cycle of chronic infection and inflammation, offering a robust strategy for Gram‐negative bacteria‐infected diabetic wound management.

## Graphical abstract

Chronic diabetic wounds are hindered by a “dual trap” of biofilm resistance and hyperinflammation. A mesoporous catalytic‐adsorptive nano‐regulator is developed to dismantle this barrier through simultaneous eDNA degradation and LPS sequestration. By silencing TLR4/9‐mediated signaling, the platform clears resilient pathogens and reprograms the immune landscape, effectively accelerating bacterial clearance and promoting tissue regeneration.

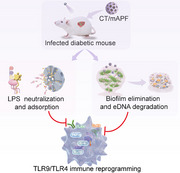

## 1 Introduction

Diabetic foot infections (DFI) represent a critical complication of diabetes and remain a key driver of non‐traumatic lower‐limb amputations [1, 2, 3]. Unlike normal acute healing, the diabetic wound bed is intrinsically compromised by a highly dysregulated immune landscape, characterized by excessive oxidative stress and the persistent accumulation of pro‐inflammatory M1 macrophages, which severely stall the transition from the inflammatory to the proliferative phase [4]. The clinical intractability of DFI is largely attributed to the biofilm, which functions as a “dual trap” by integrating structural resistance with immunological interference [1, 5, 6]. Specifically, the dense extracellular polymeric substance (EPS) matrix acts as a physical barrier that obstructs antibiotic diffusion. Concurrently, the accumulation of pathogen‐associated molecular patterns (PAMPs) within this matrix maintains a state of chronic hyperinflammation [5, 7]. This synergistic mechanism ensures that even if planktonic bacteria are cleared by conventional antibiotics, the persistent biofilm remnants and inflammatory stimuli continue to obstruct tissue repair [8]. Therefore, an effective therapeutic strategy must exceed conventional bactericidal activity; it calls for biomaterials designed for both structural biofilm disruption and localized immunomodulation to effectively resolve the cycle of chronic infection. As emphasized by recent cutting‐edge strategies in diabetic wound care, overcoming this recalcitrance necessitates the development of multifunctional nano‐engineered biomaterials capable of simultaneously dismantling structural biofilms and orchestrating localized immunomodulation to rescue the hostile diabetic microenvironment [9].

However, the recalcitrance of DFI is not merely a product of bacterial persistence but is exacerbated by a self‐fueling hyperinflammatory microenvironment [10, 11, 12]. Emerging evidence identifies extracellular DNA (eDNA) [13, 14, 15] and lipopolysaccharide (LPS) [7, 16] as the primary orchestrators of this pathological state. As a primary structural scaffold of the EPS [17, 18, 19], eDNA is released via programmed bacterial lysis or active transport [20]. Beyond its structural role, eDNA simultaneously acts as a potent damage‐associated molecular pattern (DAMP). By triggering the Toll‐like receptor 9 (TLR9) pathway, eDNA recruits immune cells into a futile, pro‐inflammatory cycle that stalls tissue regeneration [15, 21, 22, 23]. Concurrently, LPS is a major component of the outer membrane of Gram‐negative bacteria such as *P. aeruginosa* [24], which potently activates the TLR4 pathway [16, 25] through bacterial growth or antibiotic‐induced lysis and potentially escalating local inflammation into systemic cytokine storms (*TNF‐α*, *IL‐6*, and *IL‐1β*) [25]. Consequently, achieving clinical resolution requires a strategy shift: from simple bactericidal intervention to a triad‐strategy of biofilm disruption, bacterial eradication, and the active neutralization of inflammatory debris (eDNA and LPS).

In the complex microenvironment of bacterial biofilms, eDNA and LPS are functionally coupled to form a sophisticated defense system. Structurally, eDNA acts as a polyanionic scaffold that electrostatically cross‐links LPS and cationic polysaccharides [26, 27]. This molecular interplay significantly enhances the Young's modulus of the biofilm matrix, creating a robust mechanical barrier that severely restricts antibiotic penetration. Beyond structural reinforcement, eDNA contributes to drug resistance through “electrostatic sequestration.” By acting as an anionic trap, eDNA masks surface‐bound LPS and intercepts cationic antibiotics, such as aminoglycosides [28], thereby delaying their binding to the bacterial membrane and reducing therapeutic efficacy. Furthermore, this eDNA‐LPS coupling extends to the host immune response, where they drive a synergistic activation of macrophages. While LPS triggers the surface TLR4 pathway [16, 25], eDNA activates endosomal TLR9 [15, 22, 23], leading to a cascaded amplification of inflammatory cytokines via shared downstream signaling molecules like MyD88 [29]. Despite these critical interactions, most current therapeutic strategies target only a single component [30, 31], often neglecting the synergistic reinforcement between these two molecules. Consequently, there is an urgent need to develop integrated biomaterials capable of achieving dual eDNA‐LPS regulation and simultaneous TLR4/9 silencing. Such a dual‐targeting approach is essential not only to dismantle the physical barriers protecting deep‐seated bacteria but also to suppress the hyper‐inflammation responsible for tissue damage.

**SCHEME 1:**
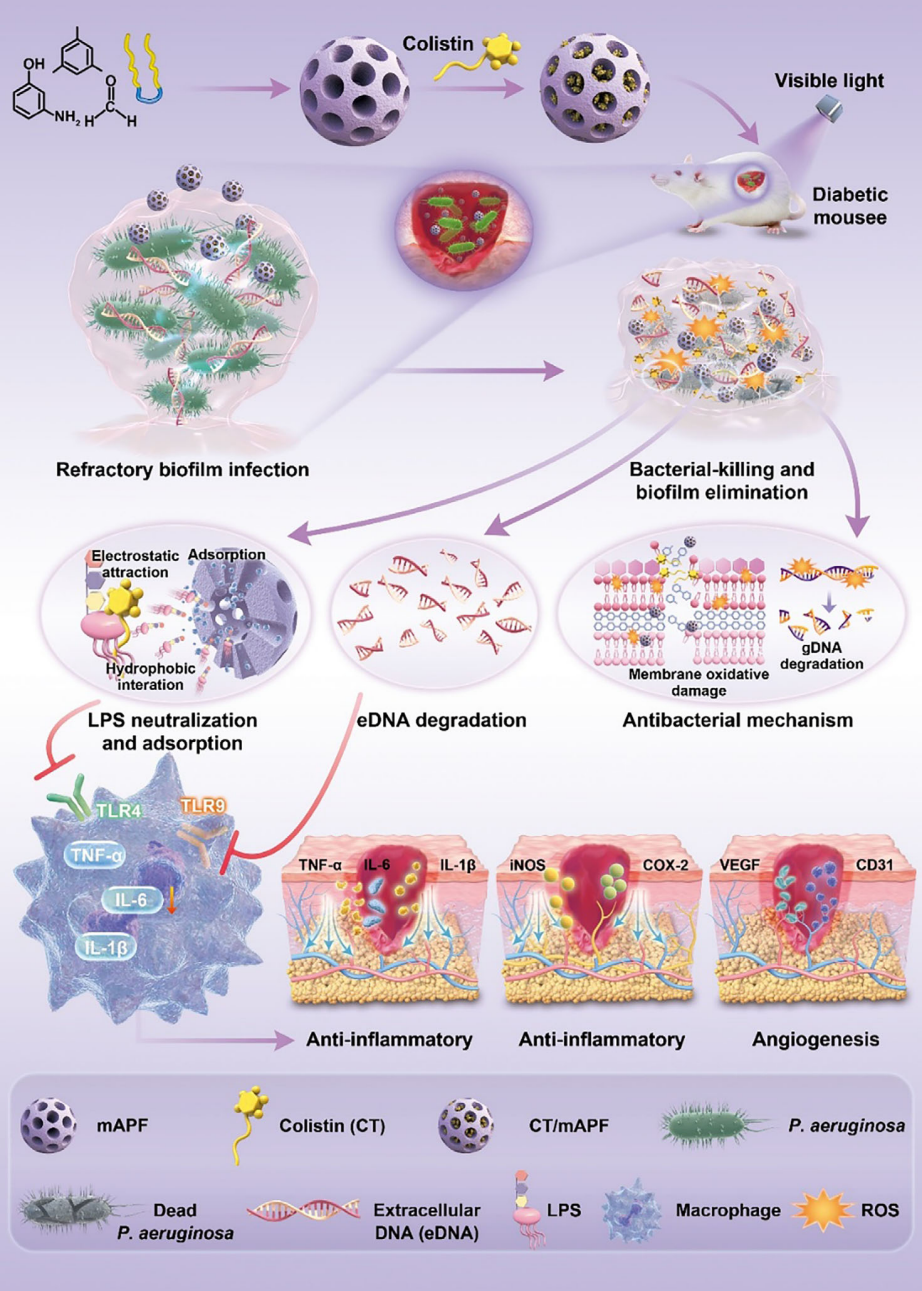
Schematic illustration of CT/mAPF nano‐regulator for accelerated wound healing in diabetic mouse.

To address the dual challenges of biofilm penetration and immune activation caused by the combination of eDNA and LPS, we developed a multifunctional mesoporous nano‐regulator to neutralize the primary molecular drivers of chronic inflammation. We hypothesize that the simultaneous degradation of eDNA and the neutralization of LPS can disrupt the structural integrity of biofilms while resolving the intrinsic inflammatory state. As illustrated in Scheme 1, the nano‐regulator is based on a mesoporous m‐aminophenol formaldehyde (mAPF) resin framework synthesized via aldol condensation and Mannich reactions under ambient conditions. This mAPF scaffold functions as a photocatalytic center, generating reactive oxygen species (ROS) upon light irradiation to initiate the oxidative degradation of eDNA. The platform is further functionalized by loading the cationic antibiotic colistin into its mesoporous channels, the polycationic nature of colistin facilitates strong electrostatic affinity for the negatively charged eDNA matrix. This leads to enhanced biofilm adhesion and subsequent localized ROS‐mediated disruption. In this system, colistin performs a dual role: it exerts direct bactericidal pressure and neutralizes free LPS through electrostatic and hydrophobic interactions. Given that colistin is a narrow‐spectrum antibiotic selectively active against Gram‐negative pathogens and LPS is exclusively confined to their outer membranes, this strategy is primarily customized to address diabetic wounds characterized by severe Gram‐negative bacterial infections, such as those propagated by *P. aeruginosa*. Simultaneously, the ROS‐induced degradation of eDNA reduces the availability of endosomal Toll‐like receptor 9 (TLR9) ligands. In vitro assays confirmed that this dual‐action platform effectively disrupts *P. aeruginosa* biofilms and suppresses the synergistic activation of the TLR4 and TLR9 pathways. In diabetic mouse models, the nano‐regulator significantly accelerated bacterial clearance and wound closure. More importantly, it successfully reprogrammed the pro‐inflammatory microenvironment toward a regenerative state, offering a robust strategy for managing complex, biofilm‐associated chronic wounds.

## 2 Results

### 2.1 Preparation and Characterization of mAPF

**FIGURE 1:**
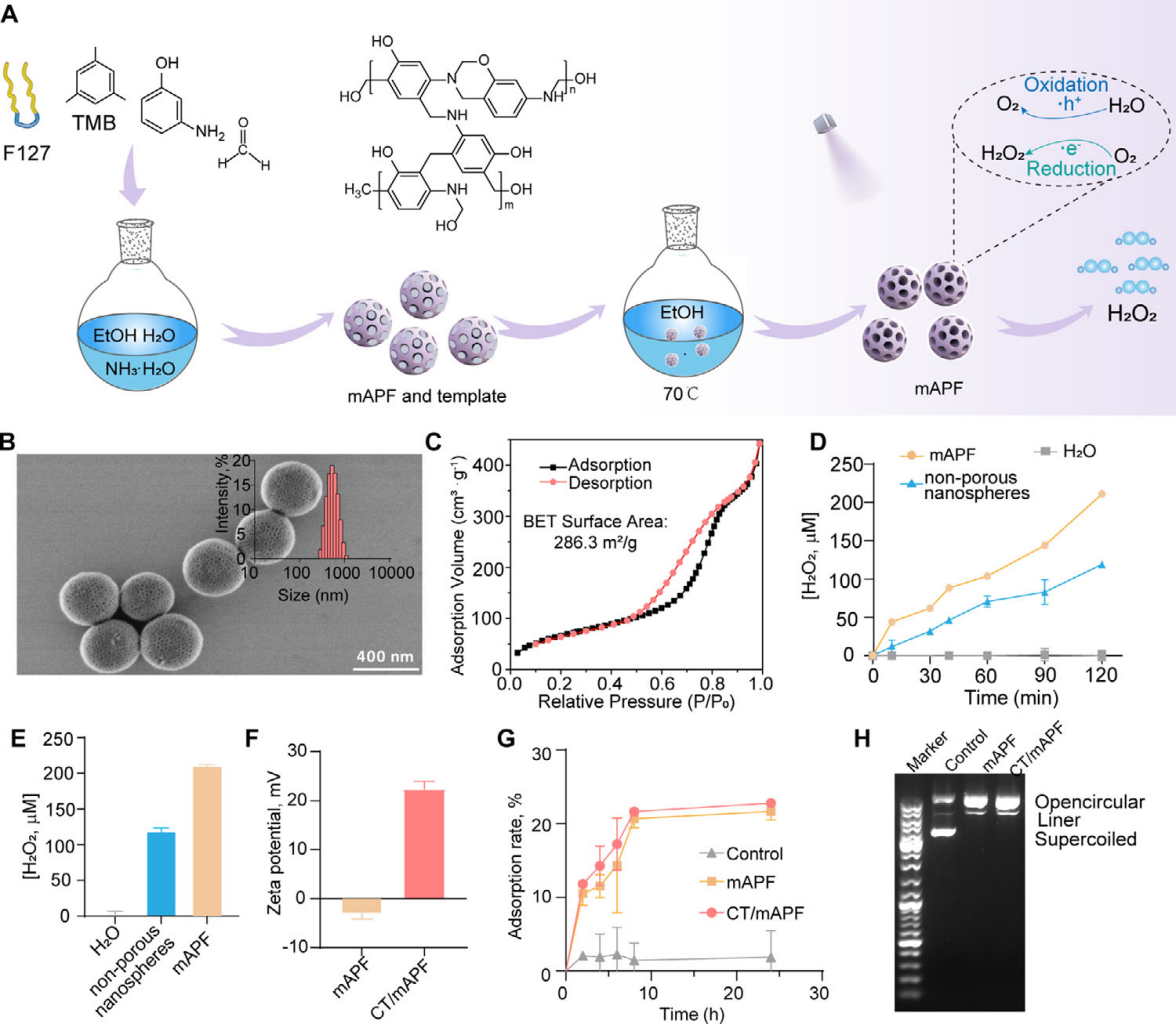
Synthesis, characterization, and antibacterial activities of mesoporous m‐aminophenol formaldehyde (mAPF). (A) Schematic illustration for the formation process of mAPF. (B) Scanning electron microscope (SEM) image and particle size distribution (inset) of mAPF. (C) N_2_ adsorption‐desorption isotherms of the mesoporous nanospheres. (D) Time‐dependent profiles of photocatalytic H_2_O_2_ generation for nonporous/mesoporous nanospheres over a 120‐min duration under light irradiation. (E) Comparative evaluation of cumulative H_2_O_2_ production at the 120‐min light irradiation. Irradiation was performed using a 300 W Xe arc lamp (λ ≥ 420 nm) with an irradiance of approximately 20 mW/cm^2^. (F) Zeta potential of mAPF before and after Colistin (CT) loading. (G) LPS adsorption rates by mAPF and CT/mAPF. (H) Agarose gel electrophoresis analysis of plasmid DNA following treatment with mAPF or CT/mAPF under light irradiation. Data are presented as mean ± s.d. (*n* = 3 independent experiments).

To address the dual requirements of targeting and regulating eDNA and LPS, a multifunctional antimicrobial nano‐regulator was developed. Given that eDNA is susceptible to degradation by hydrogen peroxide H_2_O_2_ [32], we synthesized a mAPF resin framework capable of photocatalytic H_2_O_2_ production. Uniform mAPF were first synthesized through the polymerization of m‐aminophenol and formaldehyde with Pluronic F127 as mesopore directing agent (Figure 1A). Scanning electron microscopy (SEM) images (Figure 1B, Figure S1A) reveal highly uniform mesoporous nanospheres (≈390 nm). Transmission electron microscopy (TEM) images (Figure S1B) clearly show radially oriented mesoporous channels extending from the center to the surface. Elemental analysis confirms a homogeneous distribution of C, N, and O throughout the framework (Figure S1C and D). The type II isotherms of N_2_ sorption [33] show that nanoparticle (mAPF) are mesoporous materials with surface area of 286.3 m^2^·g^−1^ (Figure 1C). The m‐aminophenol formaldehyde resin framework endows the nanoparticle the photosynthetic H_2_O_2_ production ability [34]. As shown in Figures 1D and E, the mesoporous material produced twice as much H_2_O_2_ than its non‐porous solid counterpart (Figures S2, and S3) under prolonged illumination. The efficient production of H_2_O_2_ can be attributed to the mesoporous structure, which improves substrate accessibility and promotes mass transfer, thereby endowing the nanoparticles with potent antimicrobial potential.

Subsequently, Colistin (CT), a cationic peptide with a high affinity for LPS, was successfully encapsulated into the mesoporous framework via physical adsorption to construct the multifunctional nano‐regulator, designated as CT/mAPF. The successful loading of CT was confirmed not only by the emergence of characteristic FTIR bands (Figure S4), but also by a stark shift in surface charge from ‐3.1 mV to +22.4 mV (Figure 1F), which is attributed to the cationic nature of the loaded peptide. Considering the potential hemolytic risk associated with positive surface charges, we further evaluated the hemocompatibility of CT/mAPF. As shown in Figure S5, no significant hemolysis was observed even at a concentration of 1 mg/mL, confirming its safety for subsequent applications. CT is a potent antimicrobial agent with a high intrinsic affinity for LPS (endotoxins). To evaluate the endotoxin‐neutralization capacity of the CT/mAPF nano‐regulator, adsorption assays were conducted using an LPS‐activated enzymatic cascade. As shown in Figure 1G, both the mAPF framework and the CT/mAPF nano‐regulator demonstrated effective LPS sequestration in PBS. mAPF and CT/mAPF exhibited comparable initial adsorption rates, with final sequestration efficiencies reaching 21.7% for mAPF and 22.8% for CT/mAPF over a 24‐hour period. The fact that equilibrium was not fully reached within the initial 8 h is likely due to the hindered diffusion of bulky LPS aggregates within the mesoporous channels. The initial rapid uptake is attributed to surface adsorption, while the subsequent slow phase is limited by the steric resistance encountered during intra‐particle mass transfer. To further evaluate the capacity for biofilm matrix degradation, plasmid DNA was employed as a model for eDNA to assess photocatalytic oxidative damage via gel electrophoresis. Under light irradiation, plasmid DNA treated with either mAPF or CT/mAPF showed a substantial increase in the proportion of open‐circular and linear isoforms compared to the untreated control (Figure 1H), signifying significant DNA strand cleavage. Overall, these findings demonstrate that the CT/mAPF nano‐regulator possesses dual‐functional potential, capable of both neutralizing LPS and inducing oxidative damage to eDNA.

### 2.2 Exploration of Antibacterial Capacity and Mechanism

**FIGURE 2:**
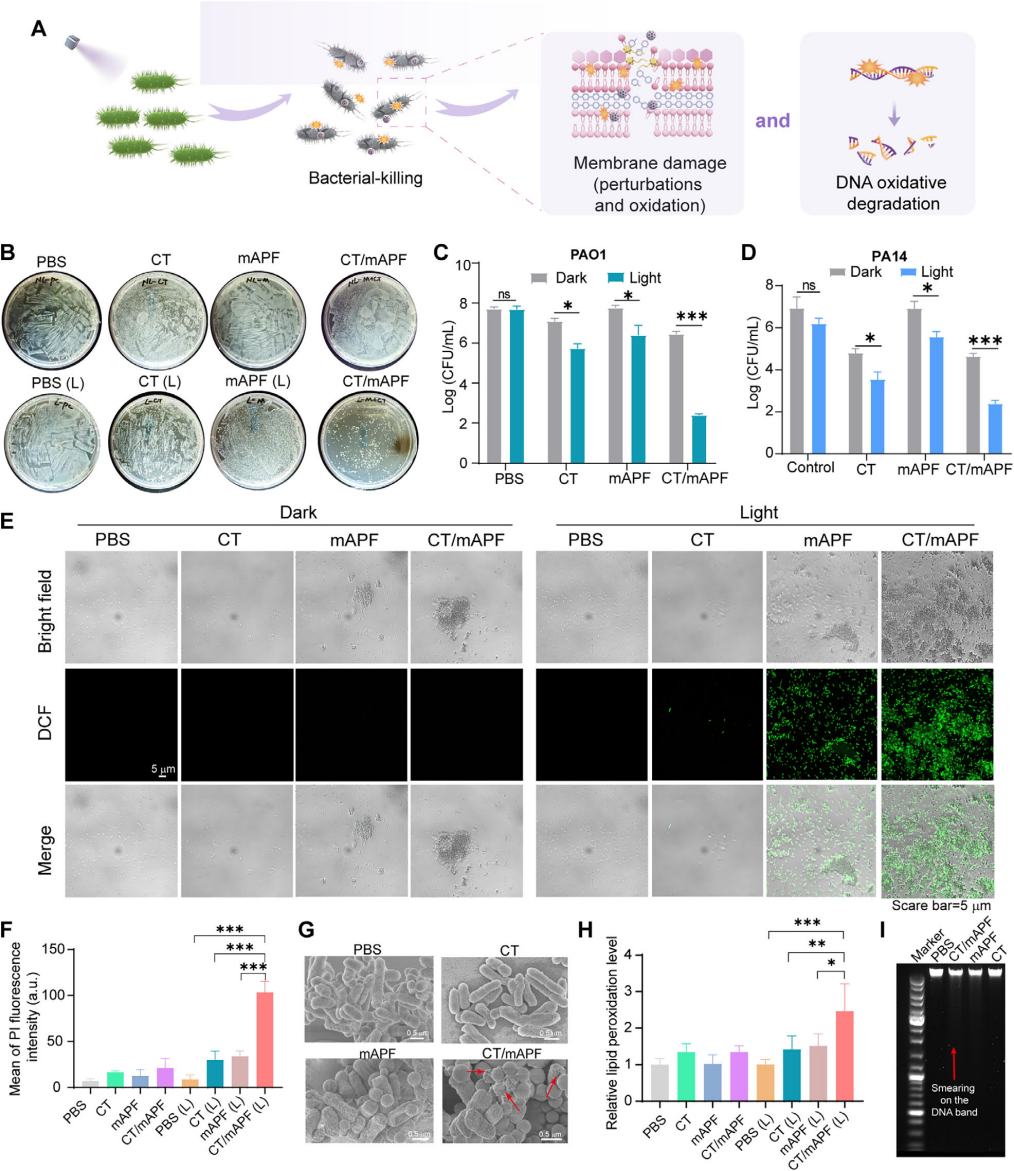
Evidences of the multi‐targeted bactericidal mechanisms of CT/mAPF: membrane disruption, lipid and genomic DNA oxidation. (A) Schematic illustration for the antimicrobial mechanism of CT/mAPF nano‐regulator. (B) Representative digital photographs of *P. aeruginosa* PAO1 bacterial colonies grown on LB agar plates with different treatments. (C) Corresponding quantitative analysis of antibacterial efficacy derived from the plate‐counting method. (D) Antibacterial activities of CT, mAPF and CT/mAPF against *P. aeruginosa* PA14. (E) Confocal laser scanning microscopy (CLSM) visualization of intracellular ROS accumulation in *P. aeruginosa* PAO1 following various treatments; ROS levels were monitored using the fluorogenic probe DCFH‐DA (green). (F) Quantitative measurement of propidium iodide (PI) accumulation under different treatment. Bacterial cell membrane disrupted by the addition of CT/mAPF under light treatment. PI is a fluorescent dye that can only penetrate compromised cell membranes, so its uptake into cells indicated membrane disruption. (G) SEM images of PAO1 cells post‐treatment with CT/mAPF under light irradiation. (H) Relative lipid peroxidation levels in PAO1, quantified via malondialdehyde (MDA) assay, for groups treated with PBS, CT, and mAPF with or without photo‐irradiation. The lipid peroxidation level of PBS‐treated group was set as 1. (I) Agarose gel electrophoresis of extracted bacterial genomic DNA stained with Gel‐red. Statistical analysis was performed using Student's *t* test: ns, **, and *** indicate *P* > 0.05, *P* ≤ 0.01, and *P* ≤ 0.001, respectively. Data are presented as mean ± s.d. (*n* = 3 independent experiments).

The antimicrobial efficacy of the composite system was initially evaluated against *Pseudomonas aeruginosa* strains PAO1 and PA14, a pathogen notorious for its propensity to form resilient biofilms that drive chronic wound recalcitrance and multi‐drug resistance [35]. As illustrated in Figure 2A, B, the CT/mAPF nano‐regulator exhibited powerful antibacterial performance under visible light irradiation, achieving eradication rates of 99.99% for PAO1 (Figure 2C) and 99.98% for PA14 (Figure 2D). These findings emphasize the platform's capacity for comprehensive clearance across distinct *P. aeruginosa* phenotypes. Notably, bare mesoporous particles alone exhibit weak bactericidal activity, indicating that mere H_2_O_2_ generation is insufficient for bacterial killing. Colistin or colistin‐loaded mesoporous particles without light‐induced H_2_O_2_ also show limited inhibition, underscoring the necessity of multi‐modal synergy. To further establish the versatility and clinical potential of this platform, we extended our investigation to a broader panel of Gram‐negative pathogens, including *Acinetobacter baumannii* and *Escherichia coli*. Notably, the CT/mAPF system maintained consistent bactericidal excellence, with eradication rates reaching 4‐log reduction (99.99%) across all tested strains (Figures S6, and S7). Importantly, In the absence of photo‐irradiation, CT/mAPF exhibited negligible cytotoxicity with an IC_50_ > 2 mg/mL (Figure S8). This dual profile of high efficacy and low dark toxicity confirms the broad‐spectrum applicability of the nano‐regulator, providing a solid foundation for the subsequent elucidation of its synergistic therapeutic mechanisms.

Having confirmed its superior antibacterial efficacy, we move on to elucidate the antimicrobial mechanism of the CT/mAPF nano‐regulator. CT/mAPF photocatalytically generates abundant intracellular ROS under visible light, as shown by intense DCF fluorescence [36], whereas negligible signal occurs without light or with the bare carrier, confirming strict light‐dependent ROS production (Figure 2E). Assessment of bacterial membrane integrity via propidium iodide (PI) staining revealed that PAO1 cells treated with the CT/mAPF group exhibited a distinct and robust red fluorescence signal (Figure 2F and Figure S9), indicating severe membrane compromise. Further visualization using scanning electron microscope (SEM) delineated significant bacterial aggregation and enhanced intercellular adhesion (Figure 2G). These morphological aberrations, primarily characterized by the collapse and disintegration of cell surface architectures, confirm the extensive physical damage inflicted by the synergistic action of photocatalytic oxidative stress and colistin‐mediated membrane destabilization. Furthermore, all photocatalytic treatment groups exhibited varying degrees of lipid peroxidation relative to the untreated control (Figure 2H). Specifically, the individual treatments of mAPF and CT colistin increased the levels of malondialdehyde (MDA), a definitive byproduct of polyunsaturated fatty acid degradation [37], to approximately 1.5‐fold and 1.4‐fold of the control, respectively. In contrast, the photocatalytic CT/mAPF group demonstrated the most significant enhancement, with MDA concentrations reaching approximately 2.3‐fold that of the control. These results highlight the superior efficacy of the CT/mAPF nano‐regulator in triggering membrane‐destructive lipid peroxidation, likely through a synergistic effect between photocatalytic ROS generation and peptide‐induced membrane destabilization. Agarose gel electrophoresis results further confirmed that after treatment with CT/mAPF, the genomic DNA bands of the bacteria exhibited significant smearing and diffusion (Figure 2I). This visual evidence confirms the occurrence of oxidative fragmentation and extensive structural damage to the bacterial genome.

**FIGURE 3:**
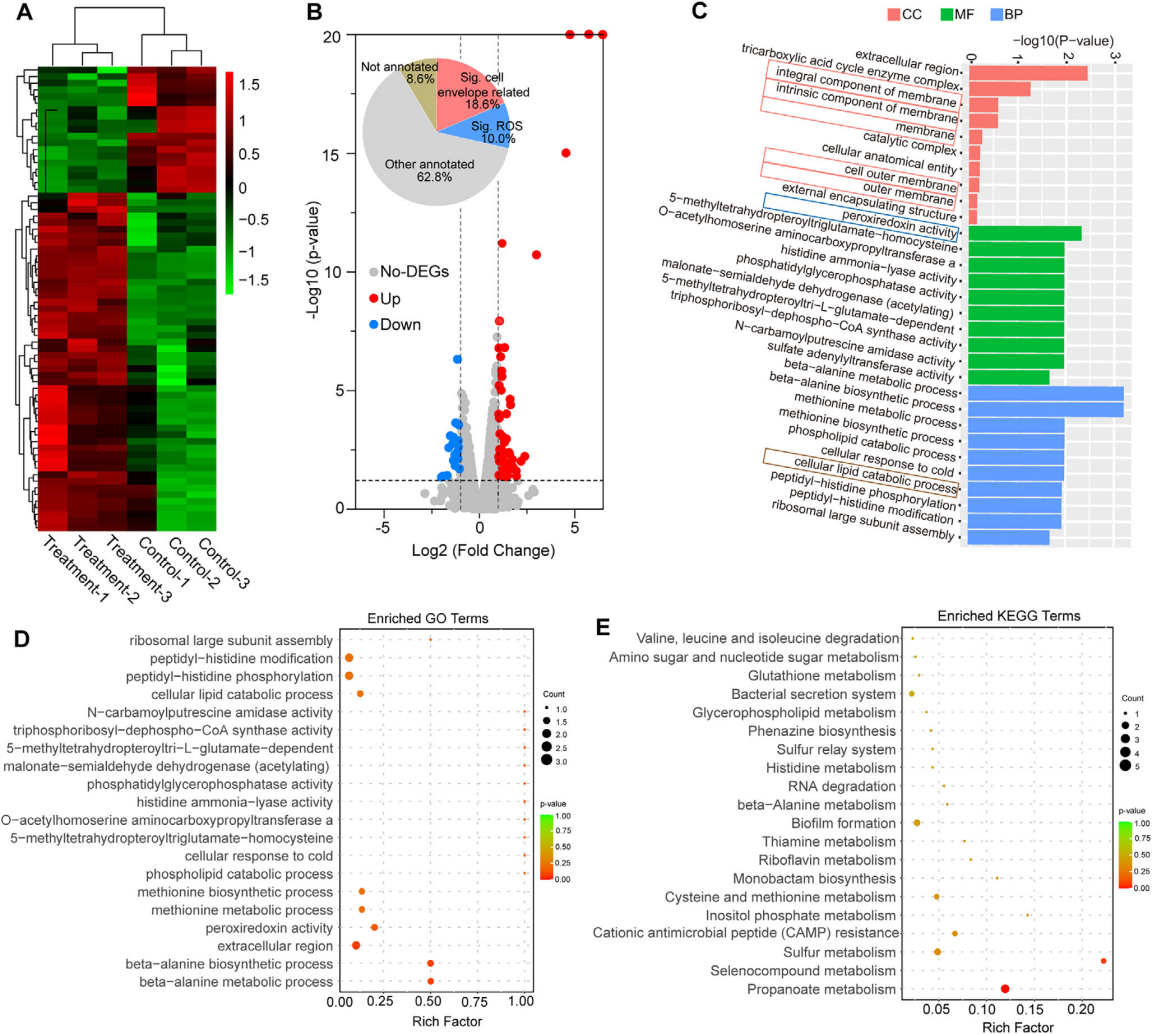
Overview of bacterial gene transcriptome sequencing outcomes. (A) Heatmap of differential expression analysis showing gene regulation changes in PAO1 treated with CT/mAPF. (B) Volcano plot of the transcriptome results using CT/mAPF ‐treated PAO1. Up‐regulated genes in red and down regulated genes in blue. log2 fold change: log2 (fold change of RPKM for a certain gene); −logP: −Log10 of *p*‐value for the log2 fold change of a certain gene. (C) Gene Ontology (GO) analysis reveling the overall genetic changes induced by CT/mAPF treatment in PAO1. All quantitative data are presented as the mean s.d. of at least 2 independent experiments. Red box: cell membrane related; Blue box: redox related; Brown box: lipid catabolic related. (D) Enrichment analysis of top 20 most significant GO terms in Biological process (BP) based on global DEGs (comprising both up‐ and downregulated genes) of PAO1 in response to CT/mAPF exposure. The size of the dot indicates the number of DEGs involved in the pathway. The color scale indicates the significance level. The rich factor is the ratio between the number of DEGs and all genes enriched in the pathway. (E) Enrichment analysis of top 20 significant KEGG pathways based on global DEGs of PAO1 in response to CT/mAPF exposure.

To elucidate the genetic mechanisms underlying the bactericidal activity of the CT/mAPF nano‐regulator, transcriptomic profiling was performed on *P. aeruginosa* (PAO1). Gene expression levels were quantified as fragments per kilobase per million mapped reads (FPKM). Clustered heatmap analysis revealed distinct transcriptional patterns between the control and CT/mAPF group (Figure 3A). Differentially expressed genes (DEGs) were identified using the thresholds of |log_2_(fold change)| ≥ 1 and a p‐value < 0.05. Comparative analysis of up‐ and down‐regulated genes revealed that over 28% of the DEGs were significantly associated with bacterial membrane integrity and oxidative stress response pathways. Notable up‐ or down‐regulated genes included those encoding outer membrane components, such as lipotoxin (*lptF*, PA3692), and transmembrane transporters, including the major facilitator superfamily (MFS) transporter (*PA2114*) and ABC transporters (*PA5094*). Conversely, genes encoding peroxidases (*PA3450*, *PA2331*) were markedly upregulated in response to oxidative challenge (Figure 3B). Furthermore, a significant downregulation of *PA2360*, a gene critical for biofilm maturation, was observed, suggesting that CT/mAPF nano‐regulator effectively interferes with PAO1 biofilm formation.

To further characterize the functional implications of these transcriptional alterations, Gene Ontology (GO) and Kyoto Encyclopedia of Genes and Genomes (KEGG) analyses were performed. GO classification categorized the DEGs into biological processes (BP), cellular components (CC), and molecular functions (MF) (Figure 3C,D). Within the BP ontology, “biological process” and “cellular process” were the most enriched terms. In the CC category, “membrane” emerged as the predominant term, corroborating the physical evidence of significant membrane structural alterations. Under MF, “catalytic activity” and “enzyme activity” were highly represented, highlighting the metabolic shift induced by treatment. These results indicate that CT/mAPF group treatment primarily targets bacterial membrane architecture, peroxidase activity, and lipid metabolism.

The KEGG enrichment bubble plot (Figure 3E) further revealed that the “bacterial secretion system,” “biofilm formation,” and “propanoate metabolism” were among the most significantly enriched pathways. These enriched terms, derived from the collective pool of both up‐ and downregulated DEGs, represent a combination of suppressed virulence mechanisms and activated stress responses. Specifically, pathways governing the bacterial secretion system and biofilm formation were predominantly downregulated due to the suppression of structural genes, whereas antioxidant and metabolic detoxification pathways were actively upregulated to counteract the photocatalytic challenge. Given that these pathways are essential for bacterial virulence, nutrient acquisition, and environmental adaptation, their disruption suggests a comprehensive failure of cellular homeostasis. Taken together, these transcriptomic shifts corroborate our mechanistic findings. They confirm that the CT/mAPF system achieves its bactericidal efficacy by severely disrupting membrane homeostasis and overwhelming the bacterial antioxidant defense network, which ultimately arrests vital cellular processes and destroy the structural integrity of *P. aeruginosa*.

### 2.3 The Anti‐biofilm Activity of CT/mAPF Nano‐regulator

**FIGURE 4:**
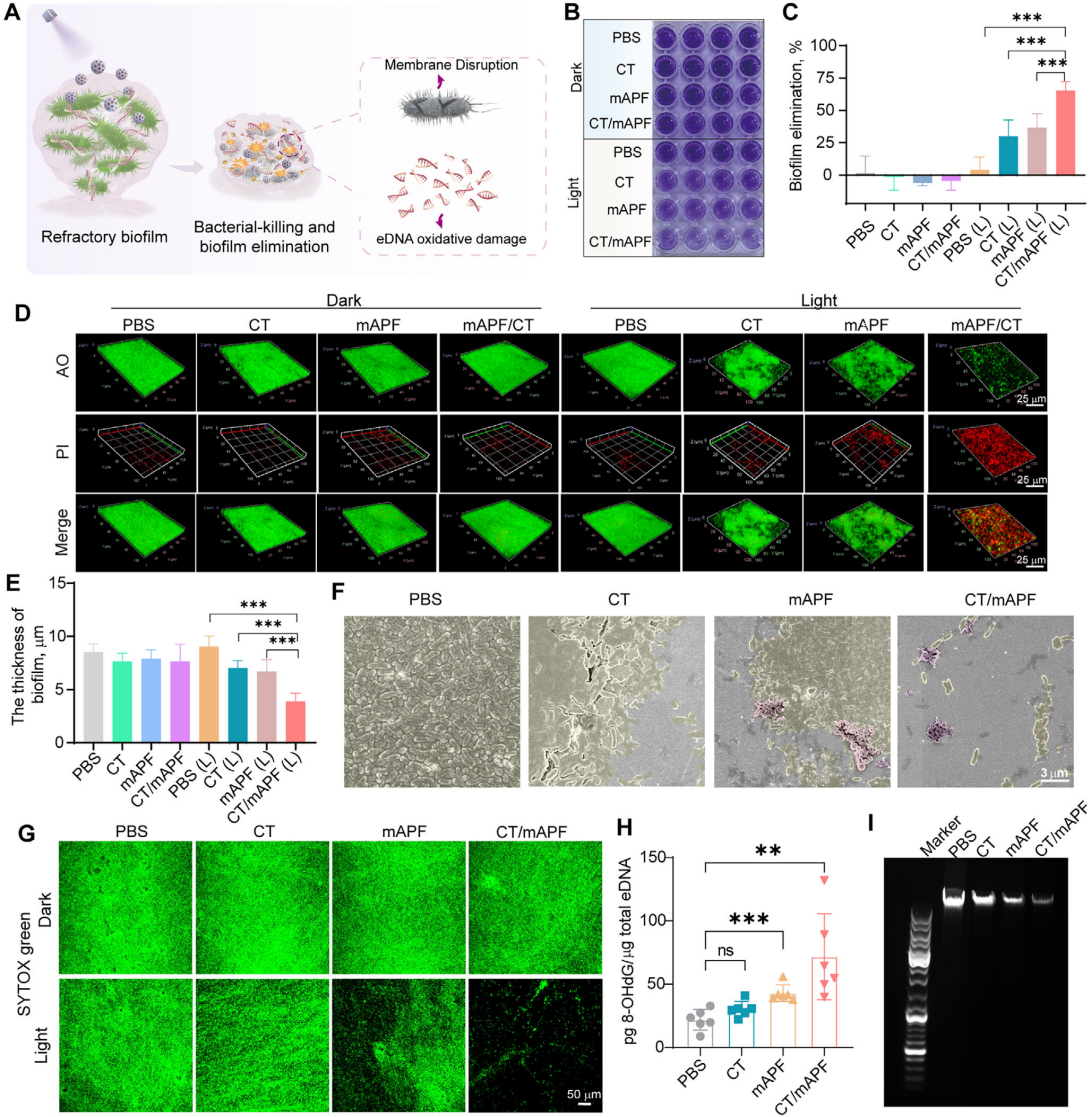
CT/mAPF effectively disrupted mature biofilms. (A) Schematic illustration for biofilm elimination of CT/mAPF nano‐regulator. (B) Representative digital photographs of crystal violet‐stained *P. aeruginosa* PAO1 biofilms following various treatments. (C) Quantitative assessment of biofilm biomass retention across experimental cohorts under dark or light‐irradiated conditions. (D) Representative 3D confocal laser scanning microscopy (CLSM) images of PAO1 biofilms; viable and dead bacteria were differentiated using AO (green) and propidium iodide (red) dual‐staining, respectively. (E) Statistical quantification of the residual architectural thickness of PAO1 biofilms derived from the CLSM analysis in (D). (F) SEM micrographs showing the micro‐morphological alterations and biomass depletion of PAO1 biofilms post‐treatment. The images are pseudo‐colored to enhance structural contrast, with the bacterial biofilm matrix highlighted in pale yellow and the CT/mAPF nano‐regulators in purple. (G) Fluorescence visualization of extracellular DNA (eDNA) within the biofilm matrix using SYTOX Green staining across different treatment groups. (H) ELISA‐based quantification of 8‐hydroxydeoxyguanosine (8‐OHdG) levels, indicating the extent of ROS‐mediated oxidative damage to the eDNA scaffold. (I) Agarose gel electrophoresis of extracted bacterial eDNA stained with Gel‐red. (ns, **, and *** indicate *p* > 0.05, *P* ≤ 0.01, and *P* ≤ 0.001, respectively, as determined by two‐tailed Student's *t*‐tests). Data are presented as mean ± s.d. (*n* = 3 independent experiments).

Employing its dual capacity to simultaneously degrade eDNA and neutralize LPS, the efficacy of the CT/mAPF nano‐regulator in disrupting mature biofilms was quantitatively assessed via a crystal violet staining assay [38]. As illustrated in Figure 4A–C and Figure S10, both free CT (28.5% reduction) and the unloaded mAPF carrier (30.6% reduction) exhibited limited antibiofilm activity, even at relatively high concentrations. In contrast, the CT/mAPF treated group achieved a substantial 65.7% reduction in biofilm biomass.

Further analysis using 3D CLSM revealed that the initial biofilm thickness (48 h post‐formation) was approximately 9.0 µm (Figure 4D,E). Following treatment with the CT/mAPF nano‐regulator, this thickness was significantly attenuated to 3.9 µm. This structural collapse was accompanied by a marked increase in bacterial mortality, as confirmed by intense PI staining (Figure 4D). SEM imaging corroborated these findings by revealing a comprehensive dispersion of the biofilm architecture. As presented in the pseudo‐colored micrographs (Figure 4F), compared to the dense, interconnected matrix in the control group (pale yellow), the CT/mAPF‐treated field exhibited a scattered accumulation of bacterial or amorphous bacterial debris alongside the deposited nano‐regulators (purple). This morphological fragmentation is consistent with the extensive oxidative cleavage of the eDNA scaffold and the subsequent physical collapse of the EPS. This disparity in performance stems from the inherent structural complexity of the biofilm, while CT and the mAPF carrier can individually target LPS and eDNA, respectively, the simultaneity of these components within the EPS matrix creates a dense, interwoven barrier. Only the synergistic interplay between ROS‐mediated matrix degradation (targeting eDNA) and the subsequent enhancement of colistin penetration (targeting LPS) can lead to a profound and extensive destabilization of the biofilm matrix.

To validate the hypothesis that the nano‐regulator induces biofilm collapse by targeting its structural scaffold, we quantified alterations in the eDNA component of *P. aeruginosa* (PAO1) biofilms. SYTOX staining revealed that upon light irradiation, pure CT elicited negligible eDNA clearance, whereas the mAPF group showed a moderate reduction. In contrast, the CT/mAPF group exhibited a pronounced decline in eDNA content (Figure 4G). Concurrently, a significant elevation of 8‐hydroxydeoxyguanosine (8‐OHdG), an established marker of DNA oxidation, was detected within the eDNA fraction of the CT/mAPF group (Figure 4H). Specifically, CT/mAPF treatment increased 8‐OHdG levels to 71.8 pg/µg, higher than mAPF, CT and the untreated control groups (24.9 pg/µg), suggesting that the nano‐regulator promotes site‐specific oxidation of guanine bases. This oxidative cleavage was further corroborated by agarose gel electrophoresis, where CT/mAPF displayed the most potent disruption of eDNA integrity (Figure 4I). The removal of eDNA by mAPF can be mechanistically linked to ROS‐mediated oxidative damage. Notably, although CT alone exhibits weak eDNA clearance capability, the combination of CT with mAPF demonstrates superior clearance efficacy compared to pristine mAPF. This can be attributed to CT's ability to capture LPS, promote biofilm collapse, cause eDNA exposure, and consequently facilitate more efficient ROS‐mediated removal. These further underscore the significance of the dual‐targeting design against LPS/eDNA.

### 2.4 In Vitro Endotoxin Adsorption and Anti‐Inflammation Properties

**FIGURE 5:**
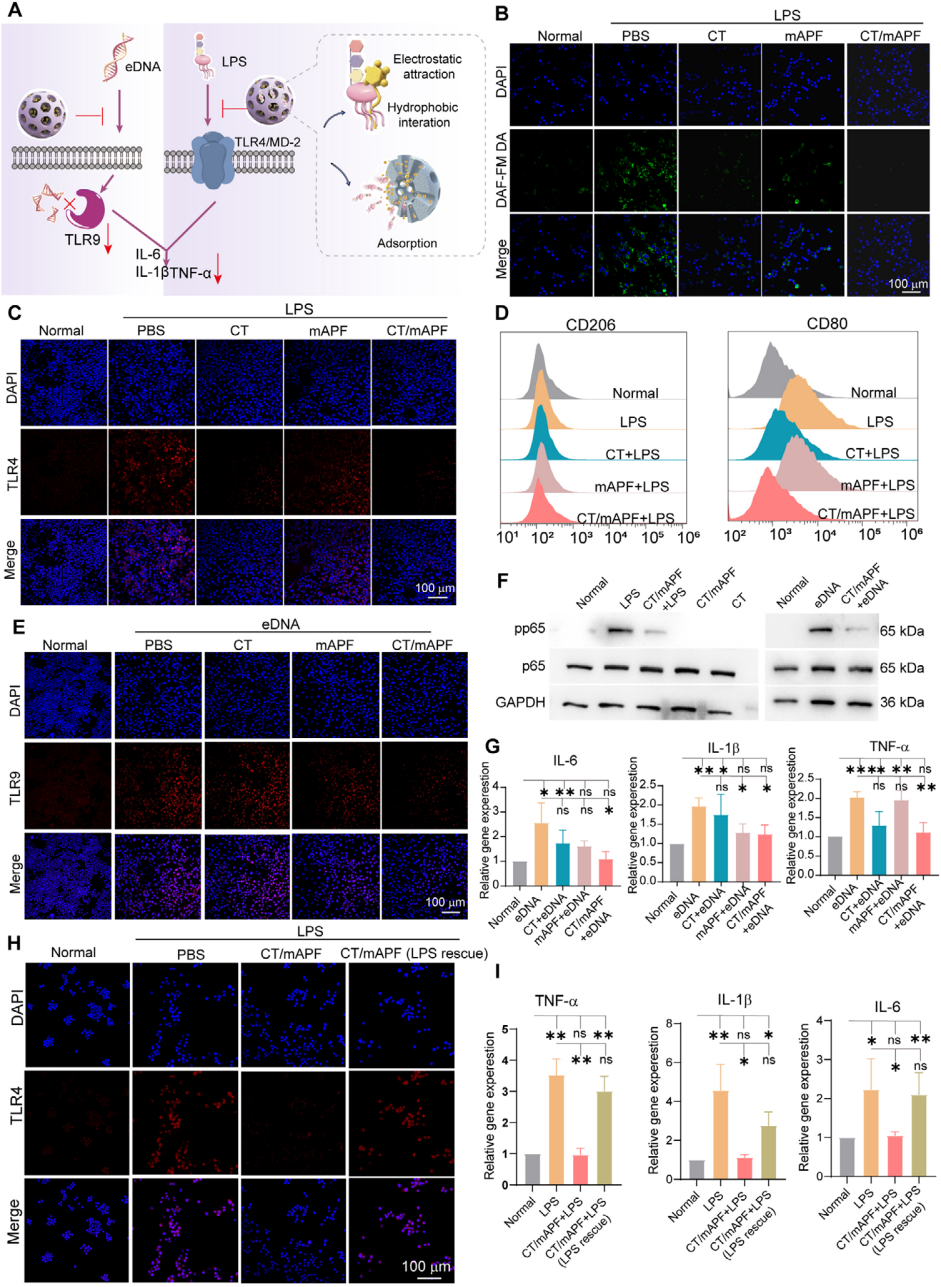
Assessment of anti‐inflammatory effects of CT/mAPF nano‐regulator in LPS/eDNA‐induced inflammatory responses in RAW 264.7 macrophages. (A) Schematic illustration of the therapeutic mechanism, highlighting the simultaneous sequestration of LPS and oxidative fragmentation of eDNA by CT/mAPF to suppress downstream inflammatory cascades. (B) Representative NO fluorescence images of DAF‐FM DA stained RAW264.7 cells after different treatments. (C) Representative immunofluorescence images of TLR4 expression in RAW 264.7 cells following various treatments for 24 h (Scale bar: 100 µm). (D) Flow cytometric profiling of macrophage polarization markers, CD80 (M1 phenotype) and CD206 (M2 phenotype) with different treatments for 24 h. (E) Representative immunofluorescence images of TLR9 expression in RAW 264.7 cells subjected to different eDNA‐mediated stimulations for 24 h (Scale bar: 100 µm). (F) Representative Western blot images of phosphorylated p65 (pp65) and total p65 in RAW 264.7 cells following indicated treatments. (G) RT‐qPCR analysis of *IL‐1β*, *IL‐6*, and *TNF‐α* mRNA expressions in RAW 264.7 cells challenged with biofilm‐derived eDNA fragments under different treatment. (H) Representative immunofluorescence images of TLR9 in RAW 264.7 cells after eDNA rescue. Scale bar: 100 µm. (I) RT‐qPCR analysis of mRNA expression levels of *IL‐1β*, *IL‐6*, and *TNF‐α* in RAW 264.7 cells following eDNA rescue. (ns, **, and *** indicate *p* > 0.05, *P* ≤ 0.01, and *P* ≤ 0.001, respectively, as determined by two‐tailed Student's *t*‐tests). Data are presented as mean ± s.d. (*n* = 3 independent experiments).

Effective antimicrobial therapy necessitates not only the direct eradication of pathogens but also the simultaneous mitigation of the inflammatory responses triggered by microbial debris, such as LPS and eDNA. Given that the distinct mesoporous architecture and the specialized affinity inherent to the CT/aminophenol‐based framework, we evaluated the dual‐functional capacity of our platform to neutralize LPS/eDNA and subsequently modulate downstream immune cascades (Figure 5A).

Prior to evaluating the therapeutic anti‐inflammatory effects, we first examined the intrinsic biological response of RAW 264.7 cells to the CT/mAPF nano‐regulator in the absence of external stimuli to exclude any direct immunomodulatory effects at sub‐cytotoxic levels. As shown in Figure S8, CCK‐8 assays confirmed that CT/mAPF maintains high cell viability with an IC_50_ exceeding 2 mg/mL under both dark and light conditions (Figure S8B). Notably, while free CT exhibited significant cytotoxicity at a high concentration (512 µg/mL), it showed negligible impact on cell viability at 4 µg/mL, which is the equivalent dose used in the CT/mAPF treatment group (Figure S8B). Furthermore, we monitored the baseline oxidative and polarization status of macrophages treated with CT/mAPF alone. Flow cytometric analysis and ROS staining (Figure S11) revealed that CT/mAPF is almost immunologically quiescent in the dark, with CD80 expression and ROS levels comparable to the PBS control. While light irradiation triggered a marginal increase in basal ROS and a slight shift in CD80+ populations, these alterations were negligible compared to the robust activation induced by pathological LPS treatment (Figures S11 and S12). These results establish that CT/mAPF is almost biologically inert at therapeutic dosages, confirming that the subsequent immune reprogramming is a specific response to PAMP neutralization rather than direct stimulation by the material components.

As one of a primary virulence factor, LPS binds to the TLR4 receptor on macrophages [39, 40, 41], triggering signaling pathways that drive the overproduction of intracellular reactive oxygen species (ROS). To evaluate the inhibitory effects of these nanomaterials on LPS‐induced ROS, RAW 264.7 cells were monitored using the fluorescent probe DCFH‐DA (Figure S13). LPS stimulation for 6 h elicited intense green fluorescence, signifying severe oxidative stress. While the unloaded mAPF yielded only a negligible reduction in fluorescence, the CT/mAPF nano‐regulator achieved the most pronounced suppression, restoring the fluorescence signal to baseline levels comparable to the control group. This suggests that the loaded colistin provides a biological blockade of the LPS‐TLR4 signaling axis, thereby suppressing the intracellular ROS burst at its source rather than through simple physical sequestration. The immunomodulatory role of these nano‐regulators was further verified by measuring nitric oxide (NO) production in RAW 264.7 cells (Figure 5B, Figure S14). LPS stimulation induced a sharp 15‐fold increase in NO levels, indicating robust activation of classical pro‐inflammatory pathways. Treatment with mAPF or free CT reduced NO production to 65.2% and 11.9% of the LPS‐stimulated group, respectively. Crucially, the CT/mAPF treatment exhibited the most profound anti‐inflammatory effect, suppressing NO levels to a mere 7% of the LPS‐challenged group. Immunofluorescence staining further confirmed that LPS stimulation significantly upregulated the expression of membrane‐bound TLR4. Conversely, in groups treated with CT or CT/mAPF, TLR4 expression remained comparable to the control, suggesting that effective LPS sequestration prevents receptor activation (Figure 5C). While LPS stimulation robustly upregulated the mRNA expression of *IL‐6*, *IL‐1β*, and *TNF‐α* (ranging from 2.68‐ to 4.32‐fold) (Figure S15), the CT/mAPF treatment effectively attenuated this response, restoring transcript levels to near‐normal cell values.

By effectively sequestering LPS, the CT/mAPF nano‐regulator significantly attenuated the pro‐inflammatory M1 polarization of macrophages. This suppression of the M1 phenotype suggests that CT/mAPF‐mediated endotoxin neutralization effectively disrupts the signaling pathways required for inflammatory activation [42]. Flow cytometric analysis revealed that LPS stimulation triggered a robust 3.4‐fold increase in CD80 fluorescence signal (M1 phenotype marker) intensity compared to the control group (Figure 5D and Figure S16). Conversely, treatment with free CT or the CT/mAPF nano‐regulator significantly curtailed this elevation, reducing the CD80+ fluorescence signal to 1.4‐fold and 0.91‐fold of the control levels, respectively. Notably, the proportion of CD206+ M2 macrophages remained largely unaffected across all experimental groups. Taken together, these findings demonstrate that while LPS challenge skews macrophages toward a pro‐inflammatory M1 lineage, the efficient neutralization of LPS by CT/mAPF effectively abrogates this polarization, thereby suppressing the overall inflammatory cascade.

Parallelly, eDNA serves as a pivotal inflammatory mediator by activating the TLR9 receptor, which recognizes extracellular bacterial DNA (particularly CpG motifs). As shown in Figure 5E, biofilm‐derived eDNA triggered high levels of TLR9 expression in untreated RAW 264.7 cells, which can be fully suppressed by CT/mAPF. This disparity likely stems from the fact that long‐fragment microbial DNA is a more potent TLR9 agonist. CT/mAPF treatment effectively induces the oxidative fragmentation of eDNA (Figure 4H,I), thereby diminishing its stimulatory capacity.

To bridge the gap between membrane receptor sequestration and downstream transcriptional inhibition, we evaluated the activation of the NF‐κB signaling pathway, a central a central hub for both TLR4 and TLR9 cascades. Western blot analysis (Figure 5F and Figure S17) revealed that while the LPS/eDNA challenge triggered a robust surge in p65 phosphorylation, the pp65/p65 ratios in groups treated with CT or CT/mAPF alone remained nearly identical to those of the untreated control. This lack of basal activation confirms that the material components are almost immunologically inert and do not independently perturb cellular homeostasis at therapeutic dosages. More importantly, when CT/mAPF was applied to the LPS/eDNA‐stimulated macrophages, it effectively intercepted the signal transduction, maintaining the pp65 levels at a near‐basal state. Consistent with observed TLR receptor downregulation, these protein‐level data demonstrate that the dual‐targeting strategy suppresses the MyD88/NF‐κB signaling axis through neutralization of initiating PAMPs.

These findings were further corroborated by RT‐qPCR analysis [43] of key pro‐inflammatory cytokines. Other experiments using eDNA extracted from biofilms confirmed this trend, eDNA from CT/mAPF‐treated groups exhibited a markedly diminished capacity to trigger inflammatory signaling compared to that from the untreated control. Specifically, the induction of *IL‐6*, *IL‐1β*, and *TNF‐α* expression was suppressed from initial levels of 1.98–2.25‐fold down to 1.09–1.24‐fold (Figure 5G), nearly mirroring the profile of healthy cells. Consistent with the eDNA quantification data, CT/mAPF manifested a robust antibiofilm effect that exceeded that of mAPF alone. This performance gap highlights the necessity of integrating the LPS‐binding capability of CT to overcome the biofilm barrier effectively.

To establish whether the anti‐inflammatory efficacy of CT/mAPF stems directly from its targeted neutralization of PAMPs, exogenous rescue assays were performed. As illustrated in Figure 5H, I and Figure S18, the therapeutic suppression of the TLR4 and TLR9 pathways was substantially reversed upon the re‐introduction of LPS or purified eDNA. Notably, the expression of downstream pro‐inflammatory cytokines exhibited a marked recovery; specifically, LPS stimulation significantly upregulated the mRNA levels of *IL‐6*, *IL‐1β*, and *TNF‐α* by 2.68‐ to 4.32‐fold relative to the CT/mAPF‐treated group, while eDNA supplementation elicited a 1.60‐ to 1.69‐fold increase in these markers (Figure S18). This pronounced restoration of inflammatory signaling validates that CT/mAPF exerts its therapeutic action through the targeted sequestration or degradation of these molecular triggers, thereby effectively abrogating the activation of TLR‐mediated cascades.

### 2.5 CT/mAPF Accelerates Wound Healing, Eliminates Bacteria, and Reduces Inflammation in Diabetic Mice

**FIGURE 6:**
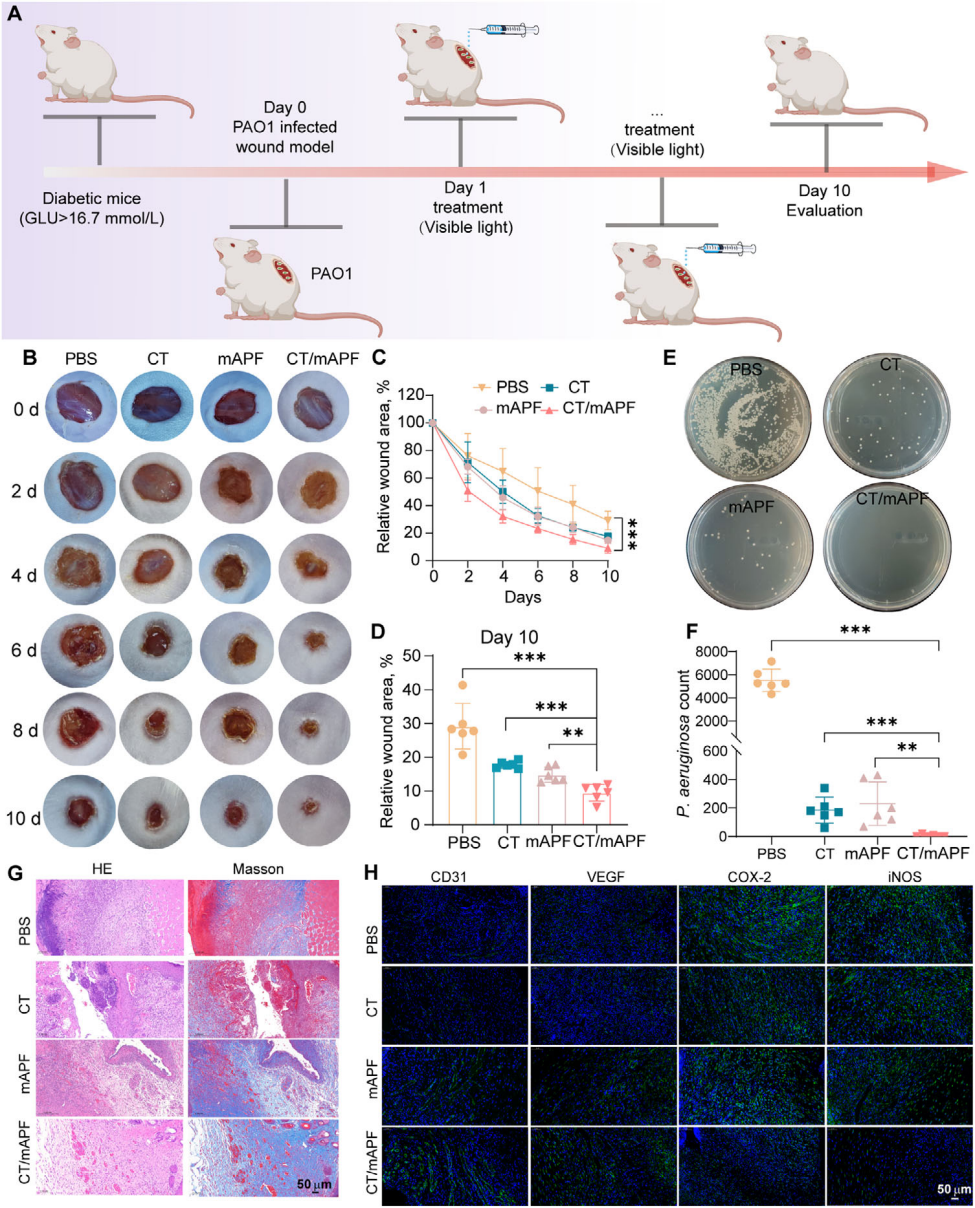
CT/mAPF nano‐regulator accelerates wound healing and reduces inflammation in diabetic mice. (A) Schematic illustration of the establishment and assessment of *P. aeruginosa* infected full‐thickness cutaneous wound in STZ‐induced diabetic mice and the subsequent therapeutic evaluation protocol. (B) Images of wound healing in mice skin at different times after different treatments. Kinetic wound contraction curves (C) and statistical comparison of the residual wound area (D) on day 10. Data are presented as mean ± s.d. (*N* = 5 animals per group). Representative images of bacterial colonies (E) and corresponding quantitative analysis of colony‐forming units (CFUs) (F) harvested from the wound beds on day 10. (G) H&E staining of the infected wound tissues (left), representative Masson staining image of mice wound (right). (Scale bar, 50 µm). (H) Representative immunofluorescence images of pro‐angiogenic markers (CD31 and VEGF) and inflammatory mediators (COX‐2 and iNOS) in regenerated wound tissues on day 10 (Scale bar: 50 µm). (Scale bar, 50 µm). (ns, **, and *** indicate *p* > 0.05, *P* ≤ 0.01, and *P* ≤ 0.001, respectively, as determined by two‐tailed Student's *t*‐tests).

Diabetic wound infection is a clinically destructive and rapidly progressing condition associated with high morbidity and mortality. To evaluate the therapeutic potential of a CT/mAPF nano‐regulator in treating *P. aeruginosa*‐infected wound model was established in diabetic mice (Figure 6A). Briefly, the diabetes was induced via daily intraperitoneal injection of 60 mg/kg streptozotocin (STZ) [44]. Blood glucose levels and body weight were monitored daily until stable hyperglycemia (blood glucose >16.7 mmol/L) was achieved. Subsequently, circular wounds were created on the dorsal skin and infected with *P. aeruginosa*, followed by treatment under different conditions with light irradiation. The wound healing process was monitored photographically (Figure 6B), and the residual wound area in each experimental group was measured daily to monitor healing progression at various postoperative stages.

Quantitative analysis of the residual wound area revealed that by day 10, the PBS‐treated control group retained a significant unhealed area (29.2%). In contrast, the free CT and mAPF treatment groups demonstrated moderate healing, with residual areas of 17.8% and 14.9%, respectively. Notably, the CT/mAPF group achieved nearly complete wound healing with minimal scarring, leaving only 9.5% of the original wound area (Figure 6B–D). To determine if this accelerated healing was linked to microbial eradication, colony‐forming unit (CFU) counts were performed on day 10. The CT/mAPF group exhibited a dramatic reduction in bacterial burden, confirming the robust anti‐infective efficacy of the synergistic strategy in vivo (Figure 6E,F).

Histological assessment via Hematoxylin and Eosin (H&E) staining was conducted to evaluate the quality of the regenerated tissue (Figure 6G). Wounds in the control group showed extensive neutrophil infiltration. In contrast, the CT/mAPF‐treated group displayed superior tissue organization, with semi‐quantitative analysis confirming a reduction in the inflammatory cell infiltration area to 0.07‐fold of the PBS control (Figure S19A). Consistently, Masson's trichrome staining demonstrated a 13.4‐fold increase in the relative collagen deposition area in the CT/mAPF group compared to the untreated baseline (Figure 6G and Figure S19B), suggesting that the nano‐regulator promotes structural tissue maturation. To assess angiogenesis, immunofluorescence staining for CD31 [45] and vascular endothelial growth factor (VEGF) [46] was performed (Figure 6H). Semi‐quantitative evaluation of the mean fluorescence intensity (MFI) revealed that the CT/mAPF treatment induced a 4.45‐fold and 4.48‐fold increase in CD31 and VEGF expression, respectively, relative to the PBS group (Figure S19C). The enhanced CD31 expression functionally corresponds to a higher density of mature microvascular structures across the wound bed, indicating accelerated vascularization.

Chronic inflammation is a primary inhibitor of dermal regeneration in diabetic wounds [47]. We further investigated the expression of key inflammatory biomarkers, including cyclooxygenase‐2 (COX‐2) and inducible nitric oxide synthase (iNOS). While PBS‐treated wounds exhibited persistently high localized expression of these mediators, the CT/mAPF group effectively suppressed the corresponding MFI of iNOS and COX‐2 to 0.28‐fold and 0.27‐fold of the control levels, respectively (Figure 6H and Figure S19C). This potent anti‐inflammatory effect is likely attributed to the simultaneous neutralization of LPS and the degradation of eDNA, which collectively intercept the TLR‐mediated inflammatory cascades and establish a microenvironment conducive to repair. Immunofluorescence confirmed that the levels of IL‐6, IL‐1β, and TNF‐α were normalized in the CT/mAPF group (Figure S20). This potent anti‐inflammatory effect is likely attributed to the simultaneous neutralization of LPS and the degradation of eDNA, which prevents the TLR‐mediated inflammatory cascade and creates a microenvironment conducive to repair.

To evaluate the biocompatibility of the nano‐regulator, the body weight of mice across all experimental groups was monitored daily throughout the treatment period. All groups exhibited a continuous and steady increase in body weight (Figure S21), indicating that the treatments did not induce acute systemic distress. Furthermore, major organs (heart, liver, spleen, lungs, and kidneys) were harvested at the experimental endpoint for comprehensive evaluation. Histopathological analysis via H&E staining (Figure S22) revealed no significant morphological alterations, inflammatory infiltration, or necrotic lesions in any of the vital organs. These findings confirm the absence of systemic toxicity and support the clinical biosafety of the CT/mAPF for the management of infected chronic wounds.

## 3 Conclusions

In summary, this study addresses the challenging clinical challenge of Gram‐negative bacterial diabetic foot infections by introducing a multifunctional mesoporous nano‐regulator, CT/mAPF, designed to resolve the recalcitrant cycle of biofilm persistence and hyperinflammation. We have successfully validated an integrated “triad‐strategy” that involves structural biofilm disruption, potent bacterial eradication, and the targeted neutralization of inflammatory debris. Central to our findings is the development of a platform capable of destroying the primary molecular drivers of the pathological wound microenvironment. Our results demonstrate that the CT/mAPF nano‐regulator effectively disrupts the biofilm's physical barrier through ROS‐mediated oxidative fragmentation of eDNA. Furthermore, by employing the high electrostatic affinity of CT for LPS sequestration, the platform achieves significant neutralization of endotoxins. This dual‐action mechanism successfully silences downstream inflammatory signaling, as evidenced by the marked suppression of TLR4 and TLR9 pathway activation and the normalization of pro‐inflammatory cytokines (*IL‐6*, *IL‐1β*, and *TNF‐α*) to normal cell levels. Both in vitro and in vivo evaluations confirm that this dual‐targeting approach not only achieves remarkable bactericidal efficacy (99.99%) but also effectively reduces inflammatory response. The significantly accelerated wound closure, enhanced angiogenesis, and matured collagen deposition observed in diabetic mouse models underscore the therapeutic potential of this system. Given its high biosafety and robust broad‐spectrum activity, the CT/mAPF nano‐regulator offers a highly effective strategy for the management of complex, Gram‐negative pathogen‐associated chronic wounds.

## 4 Experimental Sections

### 4.1 Chemicals and Biological Reagents

All the reagents and solvents were commercially available, and were used without further purification unless otherwise specified. Ammonia solution, Mesitylene, Formaldehyde, Hydrogen peroxide, Sulfuric acid (H_2_SO_4_), Acetic acid, Methanol were purchased from Sinopharm Chemical Reagent Co., Ltd. Pluronic F127 (PEO_106_PPO_70_PEO_106_) were obtained from Sigma Aldrich. Cerium (IV) sulfate and Colistin were purchased from Shanghai Aladdin Biochemical Technology Co., Ltd. Live/Dead bacterial viability kit (C2030S), Genomic DNA Mini‐Extraction Kit (Universal, Spin Column, D0063) and Gel‐Red (D0139) were purchased from Beyotime Biotech. 2’,7’‐dichlorodihydrofluorescein diacetate (DCFH‐DA) and avertin (tribromoethanol, TBE) were purchased from MeilunBio. Crystal violet was obtained from Macklin Inc., *Pseudomonas aeruginosa* (*P. aeruginosa*) PAO1, *Acinetobacter baumannii* (ATCC19606), *Escherichia coli* (*E. coli*) K12 ATCC 10798 were obtained from the American Type Culture Collection (ATCC, Manassas, VA). Water was generated using a Milli‐Q water purifier.

### 4.2 Preparation of mAPF Resins

In a typical reaction, 0.6 g of block copolymer F127 was dispersed in a mixture of 15 mL of water and 15 mL of ethanol. Then 0.5 mL of TMB and 50 µL of ammonia were added to the reaction mixture under stirring at room temperature. When the mixture became milky white, m‐aminophenol (0.5 mmol), formaldehyde solution (0.5 mmol) was added to the reaction solution.

### 4.3 Photocatalytic H_2_O_2_ Production

10 mg of mAPF was dispersed into 20 mL H_2_O. A 300 W xenon lamp (PLS‐SXE300, PerfectLight) with a cut 420 nm filter (λ ≥ 420 nm) was used as a light source. The irradiance at the surface of the reaction solution was maintained at approximately 20 mW/cm^2^. The temperature of the reaction solution was kept at 288 K. At an indicated time‐point, 1.5 mL of suspension was filtered to test the concentration of H_2_O_2_ by a traditional cerium sulfate Ce (SO_4_)_2_ titration method based on the mechanism that a yellow solution of Ce^4+^ would be reduced by H_2_O_2_ to colorless Ce^3+^. The absorbance at 316 nm was analyzed using a UV–vis spectrophotometer [48]. All photocatalysts were synthesized repeatedly and performed photoreaction for more than three times to support the conclusions.

### 4.4 Drug Loading

Physical absorption was employed to obtain CT/mAPF. Conventionally, 2.5 mg Colistin (CT) was initially dissolved in H_2_O (10.0 mL), then the mAPF (5.0 mg) was added into the solution, and the mixture was stirred at room temperature for 24 h under light‐proof conditions. The as‐prepared CT/mAPF was collected by centrifugation, and the unloaded antibiotic in the supernatant was determined by UV‐Vis or ninhydrin‐based assay (for CT) [49], and the CT entrapment efficiency (EE) of mAPF was calculated according to the following equation: EE% = (m(total antibiotic added) − m(antibiotic in supernatant))/(m(total CT added)) × 100%. m(antibiotic in supernatant) was the mass of antibiotic molecules that were not loaded on the mAPF, and m(total antibiotic added) was the mass of antibiotic added during the reaction.

### 4.5 In Vitro LPS Adsorption of CT/mAPF

The endotoxin adsorption was assessed after incubation the CT/mAPF with an LPS solution. Briefly, a nanoparticle suspension (300 µg/mL) in PBS was mixed with LPS at a final concentration of 100 ng/mL. After incubation at 37°C for 0.5, 1, 2, 4, 6, 8, or 24 h, the nanoparticles were separated from the mixture by centrifugation. The LPS concentration in the supernatant was quantified using an endotoxin detection kit (Beyotime Biotechnology Co., Ltd., China). Absorbance at 545 nm was measured with a microplate reader, and the LPS removal rate was calculated based on the ratio of residual LPS in the medium to the initially added amount.

### 4.6 Assessment of Photocatalytic Antibacterial Activity

Log‐phage *Pseudomonas aeruginosa* PAO1/PA14 (OD = 0.5) were pelleted and resuspended in PBS to prepare a working solution with initial cell density of ≈ 1 × 10^7^ CFU/mL. And the working suspension was treated with 100 µg/mL CT/mAPF for 1.5 h under lighting conditions, aliquots of the mixtures were taken out and diluted 1000‐fold using PBS, and 5 µL of each was dropped onto an agar plate. After 12 h cultivation at 37°C, colonies were count, and CFU/mL was calculated. The experiment utilizes a 300 W Xe arc lamp as the light source, with a 420 nm filter employed to eliminate light with a wavelength below 420 nm. The current running through the lamp was 12 A, resulting in a light intensity above the solution of approximately 20 mW/cm^2^. A fan was employed during the experiment to prevent the temperature from escalating.

### 4.7 In Vitro Anti‐Biofilm Assay

To test the antibiofilm activity of CT/mAPF, PAO1 (OD600 = 0.02) were cultured in 96‐well plates at 37°C for 48 h to form mature biofilms. Subsequently, the medium was removed and different samples at equivalent concentration (200 µg/mL) were added into each well and incubated for 1.5 h under lighting condition. After incubation, the medium was removed and 200 µL methanol was added for 30 min to fix biofilm. Subsequently, the methanol was removed, and the biofilms were stained with crystal violet (0.1%) for 10 min and washed with PBS. Finally, the crystal violet was dissolved in 33% acetic acid and the biomass of the biofilm was quantified by recording and calculating according to the absorbance at 590 nm. Experiments were carried out in triple.

For fluorescence microscopy imaging, mature biofilm was formed on round coverslip in 12‐well plates. After incubation for 1.5 h with 400 µg/mL samples, the medium was removed and Live/Dead bacterial viability kit (C2030S, beyotime, shanghai) was further used to evaluate antibacterial efficacy. Fluorescence microscope images were acquired using a ZEISS LSM880 Confocal Microscope. The biofilm thickness was quantified by ZEN 3.5 (blue edition) software.

For SEM imaging, biofilms formed on the round coverslip were fixed with 2.5% glutaraldehyde overnight at 4°C. The samples were then dehydrated using a series of ethanol solutions (30, 50, 70%, 90%, and 100% in Milli‐Q purified water) and sputter‐coated with gold for observation using a Hitachi S‐4800 field emission scanning electron microscope.

For eDNA analysis: The biofilms treated with different conditions were incubated with 10 µM SYTOX Green (Invitrogen S34860, USA) for 20 min in the dark. eDNA within the biofilms was visualized using a confocal microscope (ZEISS LSM880, Germany). Furthermore, 8‐OHdG (8‐hydroxydeoxyguanosine) levels in the eDNA were determined using a commercial ELISA kit (E‐EL‐0028c, Elabscience). The assay was conducted in triplicate, and the sensitivity of the ELISA kit was 0.94 ng/mL.

### 4.8 Assessment of Intrinsic Material Effects on Macrophages

To evaluate the potential direct effects of the nano‐regulator, RAW 264.7 cells (1.5 × 10^5^ cells/well) were incubated with CT/mAPF (200 µg/mL) for 24 h without LPS or eDNA stimulation. Cells were divided into dark and light‐irradiated groups (λ ≥ 420 nm, 20 mW/cm^2^, 10 min). The metabolic activity was quantified via CCK‐8 assay (1 × 10^4^ cells/well). Intracellular ROS levels were monitored using the DCFH‐DA probe, and the expression of the M1 marker CD80 and M2 marker CD206 was analyzed using flow cytometry (BD FACSCalibur). All data were compared against the PBS‐treated control group to determine the baseline activation levels.

### 4.9 In Vitro LPS Neutralization Abilities of CT/mAPF Nano‐Regulators: RAW264.7 Cells

The detoxification effect of the CT/mAPF was evaluated by measuring the intracellular expression of reactive oxygen species (ROS) and nitric oxide (NO) in macrophages. In brief, RAW264.7 macrophages were seeded in 48‐well plates at a density of 2.5 × 10^4^ cells per well and cultured for 12 h. Cells were then treated with fresh medium containing LPS (100 ng/mL) and nanoparticles (200 µg/mL). After 6 h of treatment, cells were collected, stained with 10 µM DCFH‐DA or DAF‐FM DA for 30 min, and subjected to confocal imaging analysis.

### 4.10 Immunofluorescence Evaluation

(TLR4) RAW264.7 cells were pre‐seeded (1.5 × 10^5^ cells per well) and incubated for 24 h with 100 ng/mL LPS and CT/mAPF (200 µg/mL). Cells cultured in complete medium without LPS stimulation served as the normal group (negative control). TLR4 expression was evaluated by immunofluorescence staining as follows: After washing three times with PBS, cells were fixed with 4% paraformaldehyde (PFA) for 15 min and blocked with 5% BSA for 1 h at room temperature. Cells were then incubated overnight at 4°C with a primary antibody against TLR4 (ab22048, Abcam). Following three PBS washes, cells were incubated with an Alexa Fluor 594‐conjugated goat anti‐mouse IgG secondary antibody (8890S, CST) for 1 h at 37°C. Finally, samples were mounted with an antifade mounting medium containing DAPI (P0131, Beyotime). Immunofluorescence images were acquired using laser scanning confocal microscopy (LSCM) and quantitatively analyzed with ImageJ software.

(TLR9) The samples were prepared consistent with the TLR4 assay protocols. For the immunological experiment, RAW 264.7 macrophages were stimulated with extracellular DNA (eDNA) extracted from *P. aeruginosa* biofilms that had been subjected to the respective therapeutic treatments. The purified *P. aeruginosa* eDNA was utilized at a working concentration of 10 µg/mL.

### 4.11 Macrophage Polarization Analysis

CD80 and CD206 were used as markers for M1 and M2 macrophage phenotypes, respectively. RAW264.7 cells treated with PBS, CT, mAPF, or CT/mAPF under lighting were washed three times with PBS, resuspended in stain buffer, divided into two equal portions. One portion was incubated with a PE conjugated CD80 antibody (PE‐65076, Proteintech) for 20 min at 4°C. The other portion was subjected to fixation and permeabilization before incubation with an APC‐conjugated CD206 antibody (APC‐18704, Proteintech). Samples were processed according to the manufacturer's protocol and analyzed via flow cytometry. Data were exported and analyzed using FlowJo software.

### 4.12 Quantitative Real‐Time PCR Analysis

RAW 264.7 cells were seeded in 24‐well plates and allowed to adhere for 12 h. To assess immunomodulatory activity, cells were incubated with 100 ng/mL LPS in the presence of PBS, CT, mAPF, or CT/mAPF (200 µg/mL). In another experiments, cells were stimulated with eDNA extracted from *P. aeruginosa* biofilms that had been pre‐treated with PBS, CT, mAPF, or CT/mAPF under lighting. All cultures were incubated for 24 h prior to further analysis of inflammatory markers. Total RNA was extracted with the TaKaRa MiniBEST Universal RNA Extraction Kit, following the manufacturer's instructions. The extracted RNA was converted to cDNA. The qPCR was conducted in a real‐time PCR system (QuantStudio 7 Flex, Thermo Fisher Scientific, USA) with the HiScript II One Step qRT‐PCR SYBR Green Kit (Vazyme, Nanjing). Gene expression was normalized to the expression of the housekeeping gene (*actin*). And expression levels were normalized to that of the housekeeping gene *actin*. The primer sequences for each gene were listed as Table S1.

### 4.13 Western Blotting

RAW 264.7 macrophages were seeded in 6‐well plates (1 × 10^6^ cells/well) and allowed to adhere overnight. After indicated treatments (stimulation for 1 h), cells were washed twice with ice‐cold PBS and lysed using RIPA lysis buffer supplemented with 1% protease and phosphatase inhibitor cocktails (Beyotime, China). Protein concentrations were determined using a BCA protein assay kit. Equal amounts of protein (20 µg) were separated by 10% SDS‐PAGE and transferred onto PVDF membranes. After blocking with Sealing liquid (Beyotime, China) for 1 h, membranes were incubated with primary antibodies against NF‐κB p65 (1:1000), phospho‐p65 (Ser536, 1:1000), and GAPDH (1:20000) at 4°C overnight. Subsequently, membranes were incubated with HRP‐conjugated secondary antibodies. Protein bands were visualized using an ECL chemiluminescence kit and quantified via ImageJ software.

### 4.14 In Vitro Rescue Assays

RAW 264.7 cells were seeded in 24‐well plates and allowed to adhere for 12 h. To assess the LPS and eDNA rescue activity, cells were incubated with 100 ng/mL LPS or 15 µg/mL eDNA in the presence of PBS or CT/mAPF (200 µg/mL) for 12 h to allow for initial PAMP clearance. Subsequently, the cells were re‐challenged with exogenous LPS (100 ng/mL) or purified eDNA (15 µg/mL) for an additional 12 h. Total RNA was extracted for qPCR analysis of *IL‐1β*, *IL‐6*, and *TNF‐α* expression, while protein levels of TLR4 and TLR9 were quantified via Immunofluorescence. expression levels were normalized to that of the housekeeping gene *actin*.

### 4.15 Diabetic Skin Wound Healing in Vivo

All animal experimental protocols were conducted in strict accordance with the National Institutes of Health Guide for the Care and Use of Laboratory Animals and were approved by the Institutional Animal Care and Use Committee of Fudan University (Approval No. 2024‐CHEM‐029). Furthermore, all animal experiments were authorized by the Shanghai Municipal Commission of Science and Technology.

To establish the diabetic mouse model, male ICR mice (6‐8 weeks old, Shanghai Jiesijie Laboratory Animal Co., Ltd.) were administered streptozotocin (STZ) at a dosage of 60 mg/kg through intraperitoneal (i.p.) injection for five consecutive days. The blood glucose levels of the mice were monitored using a glucometer. Mice with blood glucose levels less than 16.7 mM (random blood glucose within 2 weeks) were excluded, while the remaining mice were confirmed as diabetic mice.

Diabetic mice were anesthetized by avertin (tribromoethanol, TBE), and a round wound with a diameter of 6 mm was created on the back of the mice using scissors. Subsequently, PAO1 (2 × 10^9^ CFU) in 20 µL was dropped onto the wound to induce diabetic skin infection. the mice were divided into four groups: PBS, ROS generator, CT and CT/mAPF. After 24 h, 200 µg/kg materials were applied to the infected wound, and the mice were irradiated with a 300 W xenon lamp (PLS‐SXE300, PerfectLight) with a cut 420 nm filter for 10 min. The in vivo grouping strategy was determined based on the intended light‐activated mechanism of CT/mAPF and our preceding in vitro results, which showed little activity in the absence of irradiation. Accordingly, the animal study was designed to compare therapeutic outcomes under irradiation, while avoiding unnecessary expansion of animal numbers. Untreated PAO1‐infected mice were used as controls. The wounds were photographed daily, and the area was calculated using image J software. On day 10, representative mice from each group were euthanized by CO_2_ asphyxiation, the wounded skin was collected for histological and immunofluorescent staining, and quantitative evaluation of the bacterial burden using a standard plate counting method. Semi‐quantitative analysis of the histological and immunofluorescence images was performed using ImageJ software to determine the relative inflammatory cell infiltration area, collagen volume fraction and mean fluorescence intensity (MFI), respectively.

## Author Contributions

**J.S**.: Conceptualization, experimental design, methodology, investigation, formal analysis, project administration, writing – original draft. **Y.S**., **X.H**., **X.L**., **L.G**., **Y.W**., **T.B.Z**., **X.M.L**. and **S.W**.: Methodology, investigation, formal analysis. **D.Z**.: Supervision, methodology, formal analysis, project administration. **T.C.Z**.: Conceptualization, methodology, formal analysis, project administration, resources, funding acquisition, supervision, writing – original draft. All authors have read and agreed to the published version of the manuscript.

## Conflicts of Interest

The authors declare no conflicts of interest.

## Supporting information




**Supporting File**: advs76300‐sup‐0001‐SuppMat.docx.

## Data Availability

The data that supports the findings of this study are available in the supplementary material of this article.
