# Supplementary material for: Mesoporous Catalytic‐Adsorptive Nanoregulator Orchestrates Biofilm eDNA/LPS Disassembly and TLR9/TLR4 Immune Reprogramming to Resolve Diabetic Foot Infections
Source: Adv Sci (Weinh). 2026 Jun 26;13(52):e76300. doi: 10.1002/advs.76300 (PMC13336500; doi:10.1002/advs.76300)
Supplement: Supplementary file 1 [file ADVS-13-e76300-s001.docx]

**Supporting Information**

Mesoporous Catalytic-Adsorptive Nanoregulator Orchestrates Biofilm eDNA/LPS Disassembly and TLR9/TLR4 Immune Reprogramming to Resolve Diabetic Foot Infections

*Junfeng Song^a^, Yang Song^a^, Xirui Huang^a^, Xingjin Li^a^, Lifei Gao^a^, Yixiang Wang^a^, Tianbao Zhu^a^, Xiaomin Li^a^, Shuai Wang^a^, Tiancong Zhao*^a^, Dongyuan Zhao^a^*

a Lab of Advanced Materials, College of Smart Materials and Future Energy, Department of Chemistry, Fudan University, Shanghai 200433, China.

**Supplementary Methods**

**Electrophoretic Mobility Shift Assay**

Plasmid DNA (pCold-ctx-m-15, ~6500 bp) extracted with E.Z.N.A. plasmid Mini Kit I (Omega) was incubated with the compound of interest (300 μg/mL) under lighting for 1.5 h in PBS buffer (10 mM, pH 7.4), the mixture was run on a 1% agarose gel with Gel-Red in 1× TAE for 45 min. The DNA band shift was visualized and imaged with excitation wavelength of 480 nm using Fusion FX spectra (Vilber).

**Assessment of Photocatalytic Antibacterial Activity**

Log-phage *Pseudomonas aeruginosa* PAO1/PA14 (OD=0.5) were pelleted and resuspended in PBS to prepare a working solution with initial cell density of ~1×10^7^ CFU/mL. The working suspension was then treated with 100 μg/mL CT/mAPF for 1.5 h under lighting conditions, aliquots of the mixtures were taken out and diluted 1000-fold using PBS, and 5 μL of each was dropped onto an agar plate. After 12 h cultivation at 37 °C, colonies were counted, and CFU/mL was calculated. The experiment utilized a 300 W Xe arc lamp as the light source, with a 420 nm filter employed to eliminate light with a wavelength below 420 nm. The current running through the lamp is 12 A, resulting in a light intensity above the solution of approximately 20 mW/cm². A fan was employed during the experiment to prevent the temperature from escalating.

**Hemolysis Assay**

Fresh sheep blood was subjected to a 25-fold dilution with PBS buffer to reach a concentration of 4% blood. 500 μL of 4% blood solution containing materials at 300 μg/mL, 600 μg/mL and 1mg/mL was placed in 1.5 mL EP Tube, Then the samples were incubated at 37 °C for 2 h to allow hemolysis to take place. At the end of the incubation period, non-hemolyzed red blood cells were separated by centrifugation at 500 g for 10 min. Aliquots (100 μL) of the supernatant were transferred to a 96-well plate, and hemoglobin release was measured by absorbance at 576 nm using a microplate reader (TECAN, Switzerland). Two controls were used: an untreated red blood cell suspension in PBS as the negative control; a solution containing red blood cells lysed with 0.1% Triton-X100 as the positive control. Percent hemolysis was calculated by using the following formula: Hemolysis (%) = [(OD_576_ of the treated sample − OD_576_ of the negative control) / (OD_576_ of positive control − OD_576_ of negative control)] ×100%.

**Cytotoxicity Evaluation of CT/mAPF to RAW 264.7 and HEK293**

Cells were seeded in 96-well plates (BIOFIL, Cat. No. TCP-011-096) at a density of 1×10⁴ cells per well and incubated for 12 hours at 37 °C under a 5% CO₂ atmosphere to facilitate cell adhesion. Subsequently, the cells were treated with various concentrations of ROS generators or drug-loaded materials prepared in DMEM medium supplemented with 10% Fetal Bovine Serum (FBS), followed by a 24-hour incubation period. Cells without any compound addition served as the control group. After the incubation, the cells were washed once with 1×PBS. The culture medium was then replaced with 100 μL of fresh medium containing CCK-8 reagent, and the cells were further incubated for 0.5 hours under the same conditions (37 °C, 5% CO₂). Cell viability was determined by measuring the absorbance at a wavelength of 450 nm. Cell viability values were expressed as percentages and calculated as follows: Viability percentage = [Abs450 of treated sample) / [Abs450 of control] × 100%.

**ROS Generation Assay**

To visualize ROS production via confocal microscopy, overnight cultures of *P. aeruginosa* PAO1 (OD_600_ = 0.5) were washed three times with PBS and resuspended to an OD_600_ of 0.1. The bacterial suspension was seeded into a 24-well plate (1 mL/well) and treated with CT/mAPF for 1.5 h under light illumination. Subsequently, DCFH-DA was added to each well (final concentration: 10μM) and incubated for 20 min at 220 rpm. ROS accumulation was then captured using a confocal microscope with a 40× objective lens (DCFH-DA: Ex= 488 nm, Em= 500-550 nm).

**Effects of CT/mAPF on Bacterial Membrane Permeability**

PI was used as the fluorescent dye to evaluate integrity of bacterial membranes. A stationary-state culture of PAO1 was washed three times with PBS and then resuspended to a working concentration (OD600 = 0.1). CT/mAPF was added at 100 μg/mL and the samples were treated under lighting condition for 1.5 h. Then PI was added into the mixture with a final concentration of 50 mM for incubating 20 min at 220 rpm. Then, the samples were transferred to a glass slide for confocal imaging.

**Morphology Study by SEM Imaging**

The morphology of PAO1 before and after treatment with CT/mAPF was observed by using SEM. Log-phage PAO1 was incubated with 100 μg/mL CT/mAPF for 1.5 h under lighting condition. A bacterial suspension without any treatment was used as the negative control. Suspensions of the experiment and control groups were centrifuged (1000 g, 10 min), and the supernatants were removed. Bacteria were fixed after an overnight incubation at 4 °C with PBS containing 2.5% glutaraldehyde. Samples were washed three times with PBS and then dehydrated using a series of ethanol solutions (30, 50, 70, 90, and 100% in Milli-Q purified water). The samples were mounted on a copper tape, air-dried, and sputter-coated with gold for observation using a Hitachi S-4800 field emission scanning electron microscope. For enhanced visualization, post-acquisition pseudo-coloring of the specific SEM micrographs was performed using Adobe Illustrator software.

**MDA Level Measurement**

To determine the generation of MDA as a metabolite of lipid peroxidation in PAO1, an MDA Content Assay Kit (S0131S, Beyotime, shanghai) was utilized following the instructions provided by the manufacturer. Samples were assayed in three replicates.

**Agarose Gel Electrophoresis of Genomic DNA**

Briefly, Log-phage PAO1 (OD600=0.2) treated with samples of interest for 1.5 h with/without lighting condition. Genomic DNA of PAO1 was then extracted using the universal genomic DNA purification mini spin kit (D0063, Beyotime, Shanghai) following the manufacturer's instructions, and analyzed by the horizontal agarose gel electrophoresis. The DNA extracted was run on a 1% agarose gel with Gel-Red (2 μg/mL) in 1×TAE buffer solution for 35 min and visualized using Bio-Rad ChemiDoc™ MP Imaging System (Bio-Rad, Hercules, CA, USA).

**Transcriptome Analysis**

1. Sample preparation

The log-phase culture of PAO1 in CAMHB was subjected to treatment with 50 μg/mL of CT/mAPF under lighting conditions for 40 min. The cells were harvested by centrifuging at 3200 g for 10 min. The collected samples were then promptly frozen in liquid nitrogen

2. RNA quantification and qualification

Total RNAs obtained from replicate samples of *P. aeruginosa* PAO1 were extracted by using commercial kits, according to the manufacturer’s instructions. RNA degradation and contamination were monitored on 1% agarose gels. RNA concentrations were measured using Qubit 2.0 (Thermo Fisher Scientific, MA, USA) and Nanodrop One (Thermo Fisher Scientific, MA, USA). The RNA integrity was determined using an Agilent 2100 system (Agilent Technologies, Waldbron, Germany).

3. Library preparation

Whole mRNAseq libraries were generated using NEB Next® UltraTM Directional RNA Library Prep Kit for Illumina® (New England Biolabs, MA, USA), following the manufacturer’s recommendations. Briefly, bacterial and archaeal 16S and 23S rRNA transcripts in total RNA samples were reduced by using a Ribo-zero rRNA Removal Kit. Fragmentation was carried out using NEB Next First Strand Synthesis Reaction Buffer. The first strand cDNA was synthesized using random hexamer primer and M-MuLV Reverse Transcriptase (RNase H). During the synthesis of the second strand of cDNA, a chain-specific library was constructed by replacing dTTP with dUTP, to improve accuracy. Remaining overhangs were converted into blunt ends via exonuclease/polymerase reactions. After adenylation of the 3’ ends of DNA fragments, NEB Next Adaptor with a hairpin loop structure were ligated to be ready for hybridization. In order to select cDNA fragments of about 150-200 bp length, fragments were selected with AMPure XP beads (Beckman Coulter, Beverly, USA). Then, PCR was performed with Phusion High-Fidelity DNA polymerase, Universal PCR primers and Index (X) Primer. Finally, PCR products were purified with AMPure XP beads and library insert size assessed by using an Agilent 2100 system (Agilent Technologies, Waldbron, Germany)

4. Transcriptome sequencing

The clustering of the index-coded samples was performed on a cBot Cluster Generation System. After cluster generation, the library was sequenced on an Illumina Hiseq Xten platform and 150 bp paired-end reads were generated.

5. Data analysis

Data analysis was performed in the following steps:

a) Quality control. Raw data in fastq format were processed by Trimmomatic (v.0.36,ing to NCBI Rfam databases, to remove the rRNA sequence, by using Bowtie2 (v2.33, <https://github.com/BenLangmead/bowtie2>).
b) Reads mapping to the reference genome. Reference genome and gene model annotation files were downloaded from the NCBI genome website directly. The remaining mRNA sequences were mapped to the *P. aeruginosa* PAO1 reference genome by using Hisat2 (version 2.1.0, https://github.com/infphilo/hisat2).
c) Transcript quantification and sample relationship analysis. HTSeq-count (v0.9.1, <http://htseq.readthedocs.io/en/release_> 0.9.1/) was used to obtain the read count and function information of each gene, based on the mapping results. In order to make the expression levels of genes be comparable among different genes and different experiments, the RPKM of each gene was calculated. RPKM, Reads Per Kilobase of transcript, per Million mapped reads, is a normalized unit of transcript expression and considers the effects of sequencing depth and gene length for the read count, and is currently the most commonly used method for estimating gene expression levels. PCA (principal component analysis), correlation coefficient heat maps and expression heat maps were then used to reveal transcription relationships between all samples.
d) Differential expression analysis. Read count of each gene obtained from HTSeq-count was used for differential expression analysis. Differential expression analysis of gene expression data was performed using edgeR (v3.16.5, <http://www> bioconductor.org/packages/release/bioc/html/edgeR.html), which takes the length and number of genes into account. The resulting P-values were adjusted by using the Benjamini and Hochberg approach for controlling the false discovery rate (FDR). Genes with FDR ≤ 0.05 and |log2(fold change)| ≥ 1 were taken as differentially expressed genes, and these were used for heatmap construction.
e) Bioinformatic analysis. GO (Gene Ontology, http://www.geneontology.org) annotation analysis of differentially expressed genes was implemented by using clusterProfiler (v3.4.4, <http://www.bioconductor.org/packages/release/bioc/html/> clusterProfiler.html). Searching of sequence homologs, secondary structure prediction and protein 3D structure prediction of gene products were performed with the Protein Basic Local Alignment Search Tool (BLAST, see *J. Mol. Biol.* **1990**, *215*, 403- 410), TMHMM (see *J. Mol. Biol.* **2001**, *305*, 567-580) and Phyre2 (*Nat. Protoc.* **2015**, *10*, 845-858) servers, respectively.

***P. aeruginosa* eDNA Isolation**

eDNA was extracted from *P. aeruginosa* PAO1 biofilms according to previously established protocol. To eliminate contaminating LPS, the extracted eDNA was purified using Detoxi-Gel Endotoxin Removing Resin (Thermo Scientific, Rockford, IL, USA). This purification process yielded eDNA preparations with low endotoxin levels (<1 pg/μg DNA). Residual LPS concentrations were quantified using endotoxin detection kit (Beyotime Biotechnology Co., Ltd., China). For all subsequent experiments, the purified *P. aeruginosa* eDNA was utilized at a working concentration of 15 μg/mL.

**Agarose Gel Electrophoresis of eDNA**

Briefly, established biofilms were subjected to the designated treatments with CT, mAPF, CT/mAPF or PBS for 1.5 h under light irradiation. Following treatment, the eDNA-containing supernatants were harvested by centrifugation. The recovered eDNA was then resolved on a 1% agarose gel containing Gel-Red in 1×TAE buffer. Electrophoresis was conducted for 45 minutes, after which the gel was visualized using a Bio-Rad ChemiDoc™ MP Imaging System (Bio-Rad, Hercules, CA, USA).

**Supplementary figures:**

**Figure S1.** (A) Scanning electron microscope (SEM) image of mAPF (B) Transmission electron microscope (TEM) image of mAPF. (C) HRTEM image and elemental mapping C, N and O. (D) The corresponding pore size distribution of mAPF.

**Figure S2.** TEM image of the control non-porous nanospheres.

**Figure S3**. N_2_ adsorption-desorption isotherms of the control non-porous nanospheres.

**Figure S4.** FT-IR spectrum of mAPF, CT and CT/mAPF.

**Figure S5.** Hemocompatibility evaluation of mAPF and CT/mAPF. (A) Representative digital photographs of hemolysis assays following treatment with mAPF and CT/mAPF (1mg/mL). (B) Hemolysis percentages of red blood cells (RBCs) incubated with mAPF and CT/mAPF at varied concentrations (300 μg/mL, 600 μg/mL, and 1 mg/mL). Data are presented as mean ± s.d. (*n* = 3 independent experiments).

**Figure S6.** Antibacterial activities of CT/mAPF against *E. coli* K12. Data are presented as mean ± s.d. (*n* = 3 independent experiments).

**Figure S7.** Antibacterial activities of CT/mAPF against *A. baumannii*. Data are presented as mean ± s.d. (*n* = 3 independent experiments).

**Figure S8**. (A) Cytotoxicity of mAPF and CT/mAPF toward RAW 264.7 and HEK293 cells. (B) Cytotoxicity of mAPF, CT/mAPF and CT toward RAW 264.7 with or without light irradiation. Data are presented as mean ± s.d. (*n* = 3 independent experiments).

**Figure S9.** (A) Results of membrane permeability assay using PAO1 and propidium iodide (PI). (B) Corresponding quantitative measurement of PI accumulation under different concentrations of mAPF and CT. Data are presented as mean ± s.d. (*n* = 3 independent experiments).

**Figure S10.** Biofilm eradication efficiency as a function of (A) mAPF concentration and (B) CT concentration under visible light irradiation. Data are presented as mean ± s.d. (*n* = 3 independent experiments).

**Figure S11.** Representative ROS ﬂuorescence images of DCFH-DA stained RAW264.7 cells after LPS or CT/mAPF only for 6 h with or without light irradiation. Scale bar: 100 μm.

**Figure S12.** Effect of the material itself on macrophage polarization. Flow cytometric analysis of M1 (CD80) and M2 (CD206) surface markers in macrophages treated with CT/mAPF alone for 24 h under dark or light irradiation conditions. Data are presented as mean ± s.d. (*n* = 3 independent experiments).

**Figure S13.** Representative ROS ﬂuorescence images of DCFH-DA stained RAW264.7 cells after diﬀerent treatments for 6 h. Scale bar: 100 μm.

**Figure S14.**  Quantitative analysis of intracellular nitric oxide (NO) levels in RAW 264.7 cells stained with DAF-FM DA following various treatments. Data are presented as mean ± s.d. (*n* = 3 independent experiments).

**Figure S15.** RT-qPCR analysis of *IL-1β*, *IL-6,* and *TNF-α* mRNA expressions in RAW 264.7 cells challenged with LPS under different treatment. Data are presented as mean ± s.d. (*n* = 3 independent experiments).

**Figure S16.** Quantitative flow cytometry analysis of M1 (CD80) and M2 (CD206) macrophage polarization markers following various treatments for 24 h. Data are presented as mean ± s.d. (*n* = 3 independent experiments).

**Figure S17.** Quantitative analysis of NF-κB p65 phosphorylation. (A, B) Quantification of pp65/p65 protein ratios normalized to the untreated control group (Normal cell). (A) Signal activation profiles in macrophages treated with LPS and the corresponding rescue effect of CT/mAPF. (B) Inhibitory effects of CT/mAPF on eDNA-induced p65 phosphorylation. Note that treatment with CT or CT/mAPF alone maintains basal signaling levels, confirming the immunological quiescence of the materials. Data are presented as mean ± s.d. (*n* = 3 independent experiments).

**Figure S18.** (A) Representative immunofluorescence images of TLR9 in RAW 264.7 cells after eDNA rescue. Scale bar: 100 μm. (B) RT-qPCR analysis of mRNA expression levels of *IL-1β*, *IL-6*, and *TNF-α* in RAW 264.7 cells following eDNA rescue. Data are presented as mean ± s.d. (*n* = 3 independent experiments).

**Figure S19.** (A) Semi-quantitative analysis of the relative inflammatory cell infiltration area derived from H&E staining images. The infiltrated areas were evaluated using ImageJ software and normalized to the PBS-treated control group (expressed as fold of PBS). (B) Semi-quantitative analysis of the relative collagen deposition area derived from Masson’s trichrome staining. (C) Quantitative comparison of the mean fluorescence intensity (MFI) for pro-angiogenic markers (CD31 and VEGF) and inflammatory mediators (COX-2 and iNOS) in the regenerated wound beds. For all semi-quantitative analyses, the data were extracted using ImageJ software and normalized to the PBS-treated control group (expressed as fold of PBS). Statistical significance was analyzed using Student’s *t*-tests: ns, **, and *** indicate *P* > 0.05, *P* ≤ 0.01, and *P* ≤ 0.001, respectively.

**Figure S20.** Representative immunoﬂuorescence images of IL-6, IL-1β, and TNF-α in wound tissue treated by control and different materials at the experimental endpoint.

**Figure S21.** Mouse body weight was monitored throughout the treatment period.

**Figure S22.** Tissue immunohistology section images of mice treated with PBS, CT, mAPF and CT/mAPF.

**Table S1.** The primer sequences used in quantitative real-time PCR analysis.

| Gene name | Sequence (5'-3') | |
| --- | --- | --- |
|  | Forward | Reverse |
| *actin* | GGCTGTATTCCCCTCCATCG | CCAGTTGGTAACAATGCCATGT |
| *IL-1β* | AGATGAAGGGCTGCTTCCAAA | AATGGGAACGTCACACACCA |
| *TNF-α* | CAGGCGGTGCCTATGTCTC | CGATCACCCCGAAGTTCAGTAG |
| *IL-6* | TAGTCCTTCCTACCCCAATTTCC | TTGGTCCTTAGCCACTCCTTC |
